# Continuous high-frequency pesticide monitoring to observe the unexpected and the overlooked

**DOI:** 10.1016/j.wroa.2021.100125

**Published:** 2021-11-07

**Authors:** D. la Cecilia, A. Dax, H. Ehmann, M. Koster, H. Singer, C. Stamm

**Affiliations:** a Eawag: Swiss Federal Institute of Aquatic Science and Technology, Dübendorf, Switzerland; b Cantonal Office for the Environment, Thurgau 8510, Frauenfeld, Switzerland

**Keywords:** Plant protection products, Water quality, Legacy contaminants, High-frequency monitoring, High resolution mass spectrometry

## Abstract

Synthetic Plant Protection Products (PPPs) are a key element for a large part of today's global food systems. However, the transport of PPPs and their transformation products (TPs) to water bodies has serious negative effects on aquatic ecosystems. Small streams in agricultural catchments may experience pronounced concentration peaks given the proximity to fields and poor dilution capacity. Traditional sampling approaches often prevent a comprehensive understanding of PPPs and TPs concentration patterns being limited by trade-offs between temporal resolution and duration of the observation period. These limitations result in a knowledge gap for accurate ecotoxicological risk assessment and the achievement of optimal monitoring strategies for risk mitigation. We present here high-frequency PPPs and TPs concentration time-series measured with the autonomous *MS^2^Field* platform that combines continuous sampling and on-site measurements with a high-resolution mass spectrometer, which allows for overcoming temporal trade-offs. In a small agricultural catchment, we continuously measured 60 compounds at 20 minutes resolution for 41 days during the growing season. This observation period included 8 large and 15 small rain events and provided 2560 concentration values per compound.

To identify similarities and differences among the compound-specific concentration time-series, we analysed the entire dataset with positive matrix factorisation. Six factors sufficiently captured the overall complexity in concentration dynamics. While one factor reflected dilution during rainfall, five factors identified PPPs groups that seemed to share a common history of recent applications. The investigation per event of the concentration time-series revealed a surprising complexity of dynamic patterns; physico-chemical properties of the compounds did not influence the (dis)similarity of chemographs. Some PPPs concentration peaks led while others lagged by several hours the water level peaks during large events. During small events, water level peaks always preceded concentration peaks, which were generally only observed when the water levels had almost receded to pre-event levels. Thus, monitoring schemes relying on rainfall or water level as proxies for triggering sampling may lead to systematic biases.

The high temporal resolution revealed that the Swiss national monitoring integrating over 3.5 days underestimated critical concentration peaks by a factor of eight to more than 32, captured 3 out of 11 exceedances of legal acute quality standards (the relevant values in the Swiss Water Protection Law) and recorded 1 out of 9 exceedances of regulatory acceptable concentrations (the relevant values for the PPPs registration process).

*MS^2^Field* allowed for observing unexpected and overlooked pesticide dynamics with consequences for further research but also for monitoring. The large variability in timing of concentration peaks relative to water level calls for more in-depth analyses regarding the respective transport mechanisms. To perform these analyses, spatially distributed sampling and time-series of geo-referenced PPPs application data are needed.

## Highlights

• We produced a novel continuous, high-frequency, multi-species concentration dataset.
• High-requency captured acute toxicity exceedance during large and small rainfalls.
• We captured a large diversity of concentration patterns and peaks timing.
• Few different species unexpectedly underwent similar dynamics over rainfall events.
• Legacy pesticide dynamics indicated first-flush of pre-event water in the stream.

## Graphical abstract

**Figure:**
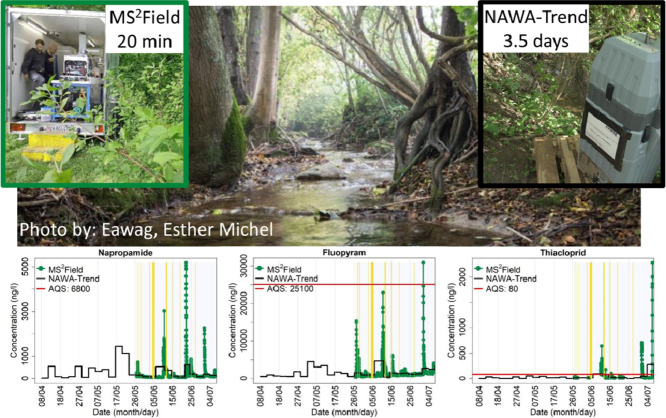

## 1 Introduction

Synthetic Plant Protection Products (PPPs) are an integral component of a large part of today's food systems at the global scale (Popp et al., 2013). Against the benefits of reduced crop losses, a number of serious negative side effects have been known for a long time. For example, PPPs and their transformation products (TPs) can reach surface water bodies, degrading their quality for both human consumption and environmental health (Gustavsson et al., 2017). The exposure of streams to PPPs varies strongly in time, with typically rather low concentration levels except during episodic events. PPPs losses depend strongly on the timing between applications and the occurrence of hydro-meteorological drivers (Leu et al., 2004; Spycher et al., 2018; Stehle et al., 2013). This holds especially true for small streams in agricultural catchments (Halbach et al., 2021). Such small streams contribute a large fraction of the entire stream length (Wohl, 2017) and play an important ecological role (Biggs et al., 2016).

A proper observation of the full exposure situation in small streams is an important basis for understanding the underlying transport mechanisms, to derive mitigation measures specifically addressing these flow processes and for designing optimal monitoring programs. However, it is a challenge to achieve a comprehensive exposure assessment (Lorenz et al., 2016). First, a large set of compounds has to be covered in the analytical effort (Curchod et al., 2020; Kiefer et al., 2021; Moschet et al., 2015; Schreiner et al., 2021; Spycher et al., 2018). There has been substantial progress in the last years in this regard and many monitoring programs cover tens to hundreds PPPs and TPs. Nevertheless, this implies a serious need of resources for being accomplished. There also exists a trade-off between temporal resolution and duration of the observation period to limit the analytical effort. The optimal monitoring strategy then very much depends on the overarching objective of the exposure assessment (Strobl & Robillard, 2008). If the focus is on testing whether chronic water quality criteria are fulfilled, time-averaged sampling is adequate and established methods of active or passive sampling can be used (Miège et al., 2015). Often there is also an interest in assessing concentration peaks against acute water quality criteria because of their ecotoxicological relevance (Liess et al., 2021). A frequently used solution for such situations is an event-driven sampling scheme. Given that many studies have revealed sharp increases of PPPs concentrations during rainfall and high discharge events, high-resolution sampling is triggered by proxies such as rainfall occurrence or discharge increases (Lefrancq et al., 2017). Sampling occurs at low temporal resolution outside of the events, when low concentration levels are expected. Despite the fact that the basic idea behind this approach is empirically well-founded, it leads to a biased exposure assessment. Any concentration fluctuations prior to the triggering event and those occurring after the sampling devices are full, remain unnoticed. The proxies also imply a certain threshold below which no data are obtained. While proxy-driven sampling can effectively reduce the number of samples, it goes with the risk of blind spots caused by mechanisms not linked to the chosen proxies. Indeed, data from a monitoring campaign of 12-hours composite samples in five small agricultural catchments (Spycher et al., 2018) over a 6-months period revealed that large rain-driven events cannot be the only cause behind elevated PPPs concentrations.

In summary, obtaining a comprehensive PPPs exposure assessment in streams is a challenge because traditional sampling and measurement techniques are limited by trade-offs between the number of analytes, temporal resolution and the duration of the observation period. To overcome these limitations, we have developed the *MS^2^Field* platform providing an autonomous workflow that combines continuous high-frequency sampling with on-site measurement with a high-resolution MSMS instrument (Stravs et al. 2021).

In this paper, we presented a comprehensive PPPs and TPs exposure dataset obtained using *MS^2^Field* in a small agricultural catchment. The study aimed at elucidating the variability of PPPs and TPs dynamics in an area characterised by a large variety of PPPs-intensive crops to study (1) how the concentration dynamics compared relative to rainfalls and water level dynamics across compounds with different physico-chemical properties and (2) to which degree acute PPPs levels were underestimated by the Swiss monitoring program based on time-composite sampling. We also addressed the degree to which the observed PPPs dynamics informed about sources and flowpaths. To these ends, we combined our concentrations dataset with public regulatory information on PPPs approval and with event-driven samples representing specific transport mechanisms (tile drains and surface runoff).

The selected catchment was known for the frequent detection of a wide range of different PPPs (Spycher et al., 2018) and belonged to the 10% of Swiss agricultural catchments classified in the top-three classes of an aggregated PPPs pollution risk potential (Koch & Prasuhn, 2021). Here, we targeted 60 compounds at 20-minutes temporal resolution continuously for 41 days encompassing different hydro-meteorological conditions (large rain events, small events and low-flow periods) during the growing season of 2019.

## 2 Methods

### Study area

**Fig. 1.**
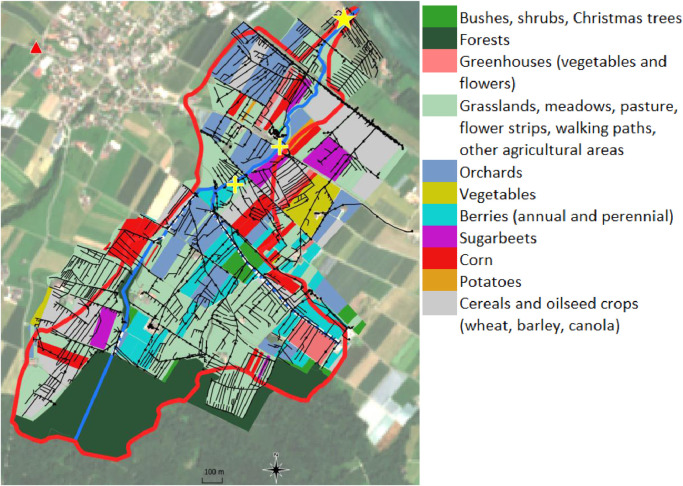
Study area. Landuse in 2019 specified in the legend at parcel level as color filled areas, weather station as red triangle, stream as open flow in blue line and as culvert in dashed white-blue line, sampling location at the catchment outlet as yellow star, drainage network as black line, maintenance holes of the drainage network in black dots, topographic catchment as red thick line, outlets “D5” downstream and “D6” upstream of the drainage network monitored by the Canton as yellow crosses (upstream runoff location “R” not displayed for protecting the privacy of the stakeholder involved). In the background, the true color image from the satellite Sentinel-2 taken on June 24^th^, 2019.

We studied a small agricultural catchment in the Swiss Plateau close to Lake Constance. A previous monitoring using 12-hours composite samples revealed exceedances of acute quality standards (AQS) and that rain-driven water level peaks lasted shorter than 12-hours, thus suggesting that higher frequency was necessary to separate peaks dynamics (Spycher et al., 2018). The topographic catchment covered about 2 km^2^. The land use in the area encompassed cereal (mainly wheat) fields (18%), orchards (17%), berries (9%), horticulture (3%), grasslands (29%), forest (19%), urban area (1%) (Source: Swiss Federal Office of Topography, Fig. 1). The altitude ranged between 400 m and 500 m with a median slope of about 3%. The sampling site was located downstream at the closure of the catchment, but sufficiently upstream to avoid influences of the lake. A water level gage managed by the cantonal environmental office took measurements at the sampling site every 15 minutes. A meteorological stations (MeteoSwiss) was located 1.8 km from the sampling site and recorded rainfall every 10 minutes. No wastewater treatment plant discharged in the stream. The catchment was drained by tile drains, which connected an estimated additional surface of 0.7 km^2^ to the stream (Source: Planimpuls.ch, Fig. 1, map retrieved after the monitoring). Given the relatively high presence of PPPs-intensive crops and farmyards connected to the stream, the catchment was ranked at high risk of PPPs pollution potential, together with 10% of Swiss agricultural streams (Koch & Prasuhn, 2021).

### Sampling campaigns

#### High-frequency short monitoring

The fully automated mobile unit *MS^2^Field* was used to collect and analyze water samples by means of high-resolution mass spectrometry with limits of quantification (LOQ) in the low range of ng/l at 20 minutes time resolution (the workflow and further technical aspects were explained in Stravs et al., (2021). Note that the sampling frequency of 20 minutes shall be considered high in general for applications in surface water. The campaign covered part of the cropping season of 2019, spanning from May 27^th^ to July 7^th^, collecting 41 days of observations. This application resulted in 2560 samples, which were analysed for 60 compounds shortlisted upon a previous screening in the stream (Spycher et al., 2018). Data gaps due to the running of quality checks and maintenance of *MS^2^Field* were not included in the 2560 samples. Details on the chemical analyses are reported in Section S1.

#### National low-frequency long monitoring (NAWA-Trend)

The sampling location was part of the Swiss National Surface Water Quality Monitoring Network (Doppler et al., 2020). In the program NAWA-Trend, 3.5-days time-composite samples were collected during the spraying season between April-July (overlapping with the *MS^2^Field* campaign) and 14-days time-composite samples otherwise. Samples were collected with a MAXX sampler with cooling unit. After the composite sample was collected, it was stored in insulated boxes with freeze-packs and transported to the laboratory in Schaffhausen (INTERKANTONALES LABOR) for chemical analysis. Details on the chemical analyses are reported in Section S2.

#### Event sampling at specific locations

During the cropping season of 2019, rain-triggered sampling devices (ISCO 6712, not refrigerating) collected event samples at two tile drain outlets and one surface runoff location. The drains were active only during wet conditions (Table 1). Each sample consisted of four pooled samples taken every 5 minutes in the first 20 minutes of water flow in the tile drain outlets and runoff location triggering the automatic sampler given the aim of the Cantonal authority to monitor the water quality during fast transport. Samples were collected within 12 hours from the activation and stored at 4 °C. In the same night, when temperatures are lower than in the day, samples were stored in insulated boxes with freeze-packs and transported to the laboratory in Belp (Interlabor Belp) for chemical analysis scheduled for next morning. Details on the chemical analyses are reported in Section S3.

### Generation of concentrations time-series within the *MS^2^Field platform*

Samples were measured by *MS^2^Field* using online solid phase extraction (oSPE)-LC-HRMS. Sample preparation and analysis are performed by a programmable autosampler (PAL RTC, CTC Analytics) and HPLC pump (Rheos 2000, Flux Instruments) connected to a high-resolution mass spectrometer (Q-Exactive HF, Thermo Fisher).

A volume of 500 – 750 µl was drawn every 20 minutes and a mix of 84 isotope-labeled internal standards was automatically spiked. The sample was enriched using custom-packed SPE cartridges (XBridge BEH C18, 5 µm) and chromatographically separated by a conventional LC column (XBridge BEH C18, 3 µm, 125 2.1 mm × 5 cm) followed by electrospray ionization (positive/negative mode switching) in fullscan mode and injection to the QExactive HF mass spectrometer (Stravs et al., 2021).

Calibration curves were performed in Evian water using the method of internal standard calibration. For the calibration, 360 PPPs and TPs together with 84 internal isotope-labeled standards of PPPs and TPs were used. To control the analyte recovery in matrix and sample carry-over, spiked samples and Evian blanks, respectively, were analysed at regular intervals during on-site measurement. Quantification was performed manually using the TraceFinder Software (Thermo Scientific) for a subset of 60 target compounds, which were selected based on the detection frequencies measured in previous more comprehensive studies of this catchment (Spycher et al., 2018). For the quantified compounds, LOQ values were in the range of 2 – 100 ng/l and the relative recovery was in the range of 48 – 139% (Section S1).

#### Data exploration

The high-temporal resolution time-series of PPPs concentrations were visually explored and analysed using RStudio 3.5.0 and the “dplyr” package.

#### Hydrological classification

The study period was divided into periods of different hydro-meteorological condition. The rationale behind the classification was to differentiate between periods when different transport processes may have been active. Large events generate discharge also by a hydrological response of the soils, while small events basically consist of runoff from hard surfaces. These hydrological differences cause different recession patterns in the hydrograph. The water level decline to pre-event conditions after large rainfalls can last hours to days, while the recession after small events is quicker. Accordingly, we distinguished between “Large events” (“large”), “Small events” (“small”) and “Dry periods” (“dry”). Based on the rainfall and water level data, a threshold of the maximum rain intensity > than 1 mm/10 minutes provided a good separation between large and small events (Tables S5-S6). Only during “large” events, stream water levels exceeded 20.6 cm. Events were split into two by a 2 hours period without rainfall. “Dry” periods referred to days of the year with no rainfall and with a calculated standard deviation of the water levels within a threshold to neglect the contribution of groundwater following rain events. In this manuscript, we will analyze the concentration time-series during large and small events. Dry periods will be the focus of a companion manuscript.

#### Patterns elucidation by positive matrix factorisation

In order to detect hidden similarities and differences in the complex set of concentration time-series, we performed a positive matrix factorisation (PMF). PMF is a multivariate receptor model approach suitable to estimate the composition of a user-defined number of contamination sources (factors) and their contribution with respect to a given multispecies dataset of concentration values (Paatero, 1994). PMF allows each data value to be weighted, thus accounting for the uncertainty in each measurement.

The input dataset is a matrix X with *n* rows and *m* columns, where *n* is the number of observations and *m* is the number of chemical species. PMF reduces the number of chemical species *m* to a user-defined number of factors *p*, each with an estimated (time) profile, by calculating the contribution *g_i,k_* of each factor (*k* from 1 to *p*) to each individual sample (*i* from 1 to *n*) according to (1):

(1) $$X=\text{FG}+E,\;\text{with}\;x_{i,j}=\sum_{k=1}^{p}f_{k,j}g_{i,k}+e_{i,j}=c_{i,j}+e_{i,j}$$

where *F* is the factor profile matrix with *p* rows and *m* columns, *G* is the factor contribution matrix with *n* rows and *p* columns, *E* is the residual (error) matrix containing the elements *e_i,j_* resulting from the estimation of *X* and *C* is the matrix of the estimated concentrations (*c_i,j_*).

The PMF solution minimizes the objective function Q, while accounting for the uncertainty (matrix *U* with elements *u_i,j_*) corresponding to (2):

(2) $$Q=\sum_{i=1}^{n}\sum_{j=1}^{m}{\left[\frac{x_{i,j}-\sum_{k=1}^{p}\left(g_{i,k}f_{k,j}\right)}{u_{i,j}}\right]}^{2}$$

For the PMF analysis, concentrations < LOQ were set to half of LOQ for the corresponding compound, where LOQ is used instead of the limit of detection (LOD) because our automated sampling does not allow for calculating a LOD. The uncertainty ($u_{i,j}$) is estimated as (Polissar et al., 1998; Yang et al., 2013):

$$\left\{ \begin{array}{c} IF\;x_{i,j}\;>\;LOQ_{j},\;u_{i,j}\;=\sqrt{{\left(a_{i,j}\times x_{i,j}\right)}^{2}+{\left(LOQ_{j}\right)}^{2}}\\ IF\;x_{i,j}\;\leq \;LOQ_{j},\;u_{i,j}\;=\frac{5}{6}\times LOQ_{j} \end{array}\right.$$

where *a* is the measurement error. *MS^2^Field* does not allow for analysing duplicate samples so as to calculate the error as the median relative percentage (Baldwin et al., 2020). Thus, we use a procedure to calculate the error specifically for each compound (details in Section S4). The error varied from 1% to 11% (Table S4).

Robust species shall be used for the PMF analysis. We used the signal/noise (S/N) ratio to assess the robustness of each chemical species for the PMF analysis, which is calculated as (3):

(3) $${\left(\frac{S}{N}\right)}_{j}=\frac{1}{n}\sum_{i=1}^{n}d_{i,j}$$

Where *d_i,j_* is a distance metric and it is calculated as:

• *d_i,j_* = 0 for *x_i,j_* ≤ LOQ*_j_*;
• *d_i,j_* = (*x_i,j_* - *u_i,j_*) / *u_i,j_* for *x_i,j_* > LOQ*_j_* .

Based on the signal to noise ratio, chemical species were classified as:

• Bad, if S/N ≤0.2;
• Weak, if 0.2 < S/N ≤ 1;
• Good, if S/N > 1.

We performed the PMF analysis by means of the freely available software EPA-PMF 5.0 (Norris et al., 2014), which also provides the environment to perform error estimation analyses (Brown et al., 2015). The software excludes bad species for the analysis and it multiplies the uncertainty for weak species by 3. Detailed information on the model are in (Norris et al., 2014).

There is no standard method to choose the number of factors a priori. Thus, we performed 20 model runs for each selected number of factor ranging from 2 to 13 (number of good chemical species minus 1). The model was run in the robust mode yielding Q_robust_ calculated as in (3 excluding concentration values corresponding to an estimated error ($e_{i,j}=\;\left|x_{i,j}-\;c_{i,j}\right|$) four times larger than the corresponding uncertainty (i.e., *e_i,j_*/*u_i,j_* > 4) to neglect possible outliers.

To assess the stability of the PMF solution we verified that Q_robust_ did not vary substantially across the 20 runs for each choice of number of factors. The model prediction error (*e_i,j_*) should be close to the estimated uncertainty (*u_i,j_*) if the research problem is adequate for a PMF analysis and the estimated uncertainty is correct (Ulbrich et al., 2009). When this happens, each term *e_i,j_*/*u_i,j_* will tend to 1 and the expected value of Q (*Q_exp_*) will tend to the number of data points in *X* minus the degrees of freedom of the weighted-least-square problem solved by PMF as *nm*_good_ - *p*(*n+m*_good_), with *m*_good_ being the number of good species (Paatero, 1994; Ulbrich et al., 2009). So, to understand the quality of the PMF solution we looked that the ratio between Q_robust_ and *Q_exp_* tended to 1. A ratio smaller than one possibly indicates overestimation of the uncertainties. We assessed the interpretability of the solutions for the factor profiles in relation with meteo-hydrological dynamics and local knowledge gained through field visits. For the selected solution, we carried a bootstrap analysis to assess the modeling error.

### Additional public data sets

We explored the use of public data sets for the identification of potential source areas given the results of the PMF analysis and the measured time-series. We retrieved the database with regulatory information compiled by the Swiss Federal Office for Agriculture (BLW database, at https://www.psm.admin.ch), which indicated the approved PPPs, the commercial products in which they can be found (together with additional active ingredients), the crop they are allowed for and the dosage. We linked this database with the land use at the parcel level to identify areas where the target compound could be potentially applied.

## 3 Results

**Fig. 2.**
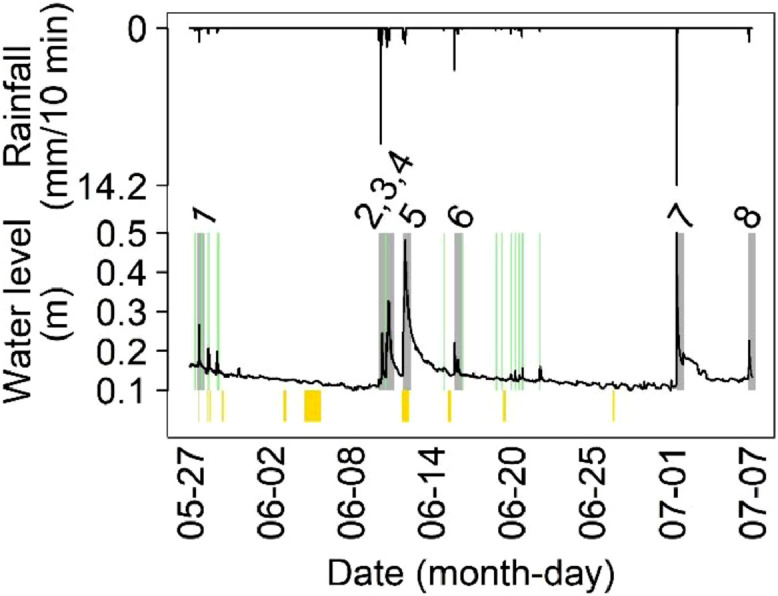
Overview of the monitoring campaign. Vertical bars in gold showed periods with data gaps due to maintenance of *MS*^*2*^*Field*. Water level shown as black line and rainfall intensity as black line on the reversed y-axis. Vertical green bars indicate small events (we highlight a period of 3 hours following the beginning of the event) and gray bars indicate large events with the number above identifying the large events (we highlight a period of 14 hours following the beginning of the event, thus events 2, 3 and 4 appear as one event on June 10^th^).

The observation period covered a wide variety of hydro-meteorological conditions. We captured eight large events lasting between 1 and 8 hours and 15 small events, of which 12 lasting less than 1 hour (Tables S5-S6), shown as gray and green bars, respectively, in Fig. 2. The total rainfall was 112.6 mm, with a maximum intensity of 14.2 mm/10 minutes and an average intensity of 1.7 mm/10 minutes during rainfalls.

Among 60 target compounds, 32 were detected above their LOQ by *MS^2^Field* (boxplots per compound in Figure S1). NAWA-Trend shared 43 out of those 60 compounds, 24 compounds exceeded their LOQ during *MS^2^Field* (full time-series in Figures S2-S5) and 10 PPPs reached their maximum concentrations in the same period (Table S7). The concentrations measured with *MS^2^Field* spanned four orders of magnitude, from few ng/l to tens of µg/l. The PPPs, grouped by class, detected with the highest concentration were the fungicide fluopyram (≈30.9 µg/l), the herbicide napropamide (≈5.1 µg/l) and the insecticide thiacloprid (≈2.2 µg/l). The 95^th^ quantile of all concentration values was ≈430 ng/l. We surprisingly measured elevated concentrations of the fungicide oxadixyl, which was withdrawn from the Swiss market in 2005. The mean concentration of this compound was ≈135 ng/l corresponding to the 4^th^ highest mean concentration in our dataset (Figure S1).

In the next subsections, we first presented the results of the PMF analyses that provided an overview on (dis)similarities between concentration time-series. Then, we showed the high-frequency concentration dynamics of the compounds used for PMF in the most relevant time window; we attempted to reveal contamination sources with the support of auxiliary data sets based on patterns similarity. After, we depicted the concentration levels measured by proxy-triggered auto samplers to highlight the role of other flowpaths for stream pollution. Finally, we summarised the *MS^2^Field* dataset in relation with the acute exposure assessment and in comparison with the NAWA-Trend data.

### 3.1 Positive matrix factorisation (PMF)

Among the 32 compounds exceeding their LOQ, we kept the 17 compounds classified with a sufficient signal to noise ratio according to (3) for the PMF analysis (Section S7). We selected the solution with six factors because the PMF model achieved good accuracies in predicting the measured concentrations, with an overall mean *R^2^* of 0.68 (Figure S6 and Table S8) and because the ratio *Q_robust_*/*Q_exp_* decreased substantially from five to six factors and only slightly from six to seven factors (Figure S7). Five species were not fit well by the model (clothianidin, R^2^ = 0.23, weak S/N ratio; fenhexamid, R^2^ = 0.36, good S/N ratio; myclobutanil, R^2^ = 0.23, good S/N ratio; simazine, R^2^ = 0.04, good S/N ratio; and thiacloprid, R^2^ = 0.28, weak S/N ratio). To obtain good model performance also for these compounds, model complexity would have to be substantially increased (Figure S6).

**Fig. 3.**
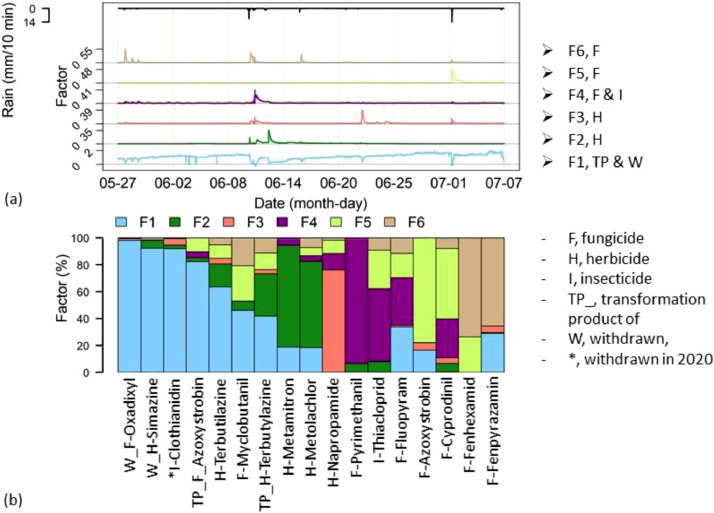
Results of the PMF. Top panel, from the bottom up, time profiles of the concentrations of each factor in every sample (matrix G) and the rainfall shown as top black line. Bottom panel, fingerprints of PPPs in every factor (matrix F). Fingerprints are indicated in the legend by the identifier “F” followed by their number. The short name for the substances in the x-labels starts with “F-” for fungicides, “H-” for herbicides, “I-“ for insecticides, “*I-“ for the later withdrawn insecticides, “W” for withdrawn, “TP-F” for fungicidal transformation product or “TP-H” for herbicidal transformation product.

The six factors of the final PMF model split in two groups: one factor that represented dilution during rainfalls and five factors that characterised different event-driven dynamics (Fig. 3a). As expected for legacy contaminants, Factor 1 contribution to the measured concentrations of the legacy fungicide oxadixyl achieved nearly 99% (purple bar in Fig. 3b). Factor 1 was predominant for simazine and other non-legacy compounds, indicating that the observed concentration dynamics during the study period were hardly affected by recent applications. The other five factors were all closely tied to rainfall events. However, they differed in the degree to which they responded to different events. Only Factor 6 showed a response to rainfall in late May; all factors except factor 5 were influenced by the events of 10 – 12 June, and the major response to the last event was observed for factor 5 (Fig. 3a). Likely, the six factors represented the contribution of different sources, activated by different events, where each event mobilised different PPPs that had recently been applied. Closer inspection revealed differences also within major events (i.e., 10 - 12 June), when the factors differed in magnitude and timing. This suggested different travel times of the compounds depending on the location within the field where they were applied and possibly due to different transport mechanisms linking the areas of PPPs application with the stream.

Interestingly, after filtering the results by enforcing a factor- and PPP-specific fingerprint contribution to the measured concentration ≥ 25%, we discovered that the factors separated classes of PPPs (Fig. 3b). The insecticide thiacloprid represented an exception because it composed factor 4 together with fungicides.

### 3.2 Contaminant concentration dynamics

#### Large events

**Fig. 4.**
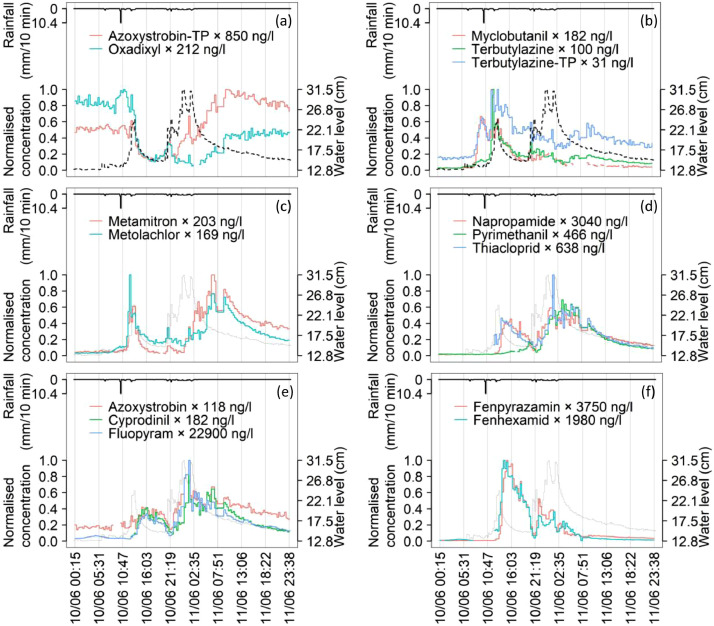
Large events between June 10^th^ and 11^th^. The legend reports the maximum concentrations of each PPPs achieved during the event; normalised concentration values are represented on the y-axis. Water level as thick black dash line in (a) and (b) and thin line for readability from (c) to (f). Rainfall as black line on the reversed y-axis. Compounds in (a) and (b) were in factor 1 of the PMF, those in (c) in factor 2, napropamide in (d) in factor 3, pyrimethanil and thiacloprid in (d) in factor 4, those in (e) in factor 5 and those in (f) in factor 6.

The PMF highlighted the largest mobilization of the most compounds during the large events 2, 3 and 4. Thus, we depicted in Fig. 4 the time-series of the selected PPPs and TPs during these consequent large events. In general, the same PPP showed different concentration patterns (peak timing and duration) in the different events. Also, some compounds showed identical dynamics within events despite having very different physical-chemical properties (Table 2). This indicated that such properties played a negligible role in the concentration patterns as compared to the possible sharing of a similar source or flowpath. The overview on measured patterns was generalizable to other events depicted in Figures S10-S13.

**Table 1.** Overview of the sampling campaigns in 2019.

Campaign	Start date	End date	Sampling site	Sample type	Temporal resolution
*MS^2^Field*	27^th^ of May	7^th^ of July	Outlet	Grab sample	20 min
NAWA Trend	7^th^ of January	23^rd^ of December	Outlet	Time composite	3.5 days (1^st^ of April to 5^th^ of August)/14 days (otherwise)
Spatial campaign 2019	5^th^ of June; 10^th^ of June; 11^th^ of June; 12^th^ of June; 1^st^ of July	5^th^ of June; 10^th^ of June; 11^th^ of June; 12^th^ of June; 1^st^ of July	Tile drain Downstream (D5)	Event composite	First 20 minutes since the triggering of the autosampler
	10^th^ of June; 11^th^ of June; 12^th^ of June; 18^th^ of June; 1^st^ of July	10^th^ of June; 11^th^ of June; 12^th^ of June; 18^th^ of June; 1^st^ of July	Tile drain Upstream (D6)		
	11^th^ of June; 12^th^ of June; 1^st^ of July	11^th^ of June; 12^th^ of June; 1^st^ of July	Runoff Upstream (R)

**Table 2.** Physical and chemical properties of the five analysed compounds retrieved from the PPDB (Lewis et al., 2016).

Name	LogP (-)	DT50Soil lab (days)	DT90Soil lab (days)	Kfoc (l/kg-OC)	DT50Photodegradation (days)	DT50Sediment (days)	DT50Water (days)
Azoxystrobin	2.5	84.5	363.3	423	8.7	205	6.1
Cyprodinil	4	53	120	2277	13.5	142	12.5
Fluopyram	3.3	309	723	278.9	21	1077	20.5
Napropamide	3.3	308	1000	885	1.5	316	28
Thiacloprid	1.26	0.88	4.3	615	NA	14.8	1000

In Fig. 4(a), we highlighted the unexpected similarity between the TP of a fungicide azoxystrobin-TP and the legacy fungicide oxadixyl, both present in factor 1 in the PMF. Noteworthy was the concentration increase in the rising limb of the water level, while dilution occurred afterwards. This suggested that pre-event water reached the outlet faster than event water. The difference in historical use of the compounds emerged at the end of the second event, when oxadixyl concentrations halved and azoxystrobin-TP ones doubled compared to their initial concentrations. The compounds in Fig. 4(b) were still grouped in factor 1. Here, we had the fungicide myclobutanil and the TP of the herbicide terbutylazine that showed the earliest peaks among all compounds, with identical dynamics in the first peak. The dynamics suggested that the compounds likely came from the same source. The BLW database indicated that terbutylazine was mainly approved for corn, while myclobutanil was approved for many uses but corn; the only shared use in the catchment was on pome fruits (approval until December 2020), which were indeed proximal to the catchment outlet (orchards in blue in Fig. 1).

Fig. 4(c) depicts the herbicides metolachlor and metamitron both predominantly grouped in factor 2. Their only shared use was for sugarbeets, which may explain the identical patterns in the second rain event. In the first rain event, metolachlor showed more similar dynamics to terbutylazine, and as a matter of fact, their only shared use was for corn where they can even be applied in the same commercial product.

Fig. 4(d) depicted together the PPPs predominantly grouped in factors 3 and 4. These compounds showed very similar dynamics only in the second rain event, matching positive and negative variations in concentrations at the 20 minutes time scale. These patterns possibly indicated the mobilization from different sources in the first event, but from the same sources afterwards. Again, the only shared use for all three PPPs was for strawberries, which were more distant from the catchment outlet.

Fig. 4(e) showed identical dynamics for the fungicides azoxystrobin, cyprodinil and fluopyram despite the concentrations differed by two orders of magnitude. These PPPs shared eleven different possible uses, which hindered from narrowing down the attention on few possible sources. Still, the PPPs equally present in factors 4 and 5 (cyprodinil, fluopyram, napropamide and thiacloprid) shared the use for strawberries only.

Finally, Fig. 4(f) displayed the similar dynamics of the fungicides fenhexamid and fenpyrazamin, with the former having many potential uses as compared to the latter; however, they only shared the use for strawberries.

#### Small events

**Fig. 5.**
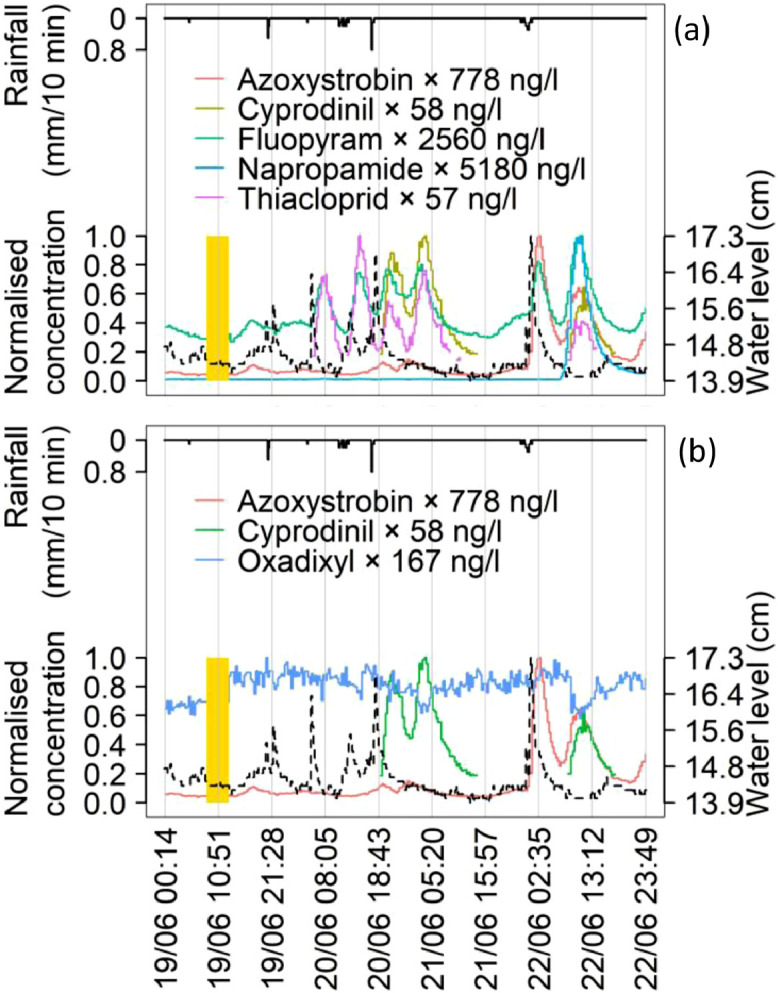
Time-series of the PPPs shown in Table 2, plus oxadixyl as groundwater “tracer” for the small events between June 19th and 22nd. The legend reports the maximum concentrations of each PPPs achieved during the event; normalised concentration values are represented on the y-axis. Concentrations below LOQ not depicted. Water level as black dash line and rainfall as black line on the reversed y-axis. Vertical bars in gold show periods with data gaps due to maintenance of *MS*^*2*^*Field*.

Fig. 5(a) showed the time-series of PPPs with similar patterns but with different physical-chemical properties (i.e., those in Table 2) during the series of small events that occurred between June 19^th^ and June 22^nd^. We observed highly dynamic and complex patterns, with concentration peaks lagging water level peaks. The data highlighted that some compounds followed identical dynamics also during small events (e.g., cyprodinil and thiacloprid). Again, we noticed long delays between rainfalls and concentration peaks (e.g., the second peak of cyprodinil occurred seven hours later than the previous small event). The last small event had a maximum intensity of 0.3 mm/10 minutes. Still, azoxystrobin showed a first peak of almost 800 ng/l. Similar to the antecedent small event, we measured a second peak of azoxystrobin after a 8 hours delay, together with the other three PPPs. Surprisingly, napropamide achieved its highest concentration of almost 5200 ng/l starting from a precedent condition of about 50 ng/l. Fig. 5(b) shows that oxadixyl, used as groundwater “tracer”, underwent dilution in the lagged peaks in the last two small events. The lagged peaks may come from distant sources/slow flowpaths not contaminated with oxadixyl.

### 3.3 Concentrations in tile drains and surface runoff

**Fig. 6.**
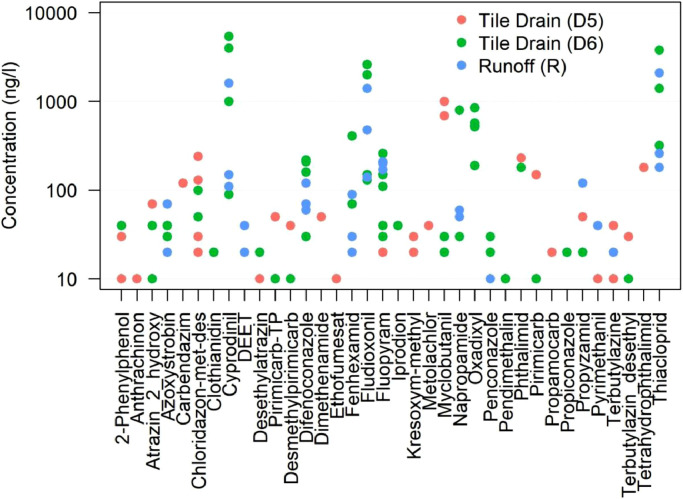
Concentration values of the measured PPPs and TPs in the drainage system and surface runoff site during the *MS*^*2*^*Field* campaign in 4 different events for D6 and R and in 5 different events for D5. Chloridazon-met-des corresponds to Chloridazon-methyl-desphenyl and Pirimicarb-TP to Desmethylformamidopirimicarb.

During the *MS^2^Field* campaign, concentrations in two tile drains “D5” downstream and “D6” upstream and one upstream surface runoff sampling point “R” ranged from 10 ng/l (the minimum LOQ) to 1000 ng/l at D5, to 5400 ng/l at D6 and to 2100 ng/l at R (Fig. 6). Six compounds measured at high concentrations in either of these three sites were also measured at high concentrations in the stream (i.e., cyprodinil, fluopyram, myclobutanil, napropamide, oxadixyl and thiacloprid). Concentrations were higher outside the *MS^2^Field* campaign, when fluopyram reached 14,400 ng/l at D5 in September and cyprodinil reached 7300 ng/l at R in August (Figure S14).

The data from these sites did not reveal clear differences in concentration levels between tile drains outflow and surface runoff. This similarity precluded to draw conclusions about the relevance of specific flowpaths for the overall concentration dynamics in the stream simply based on concentration levels. Moreover, in the event-samples at D6, oxadixyl was measured at 850 ng/l on June 10^th^ and 520 ng/l on June 11^th^, whereas its concentrations at the catchment outlet ranged between 20–212 ng/l on June 10^th^ and 6 (LOQ)-110 ng/l on June 11^th^. If we assumed that the concentrations at D6 represented the maximum peaks, then we could expect at least a 4-fold dilution in compounds’ concentrations measured at this location in the event-based sample to the catchment outlet. The short monitoring (20 minutes long) and the lack of detailed PPPs spraying records hindered disentangling the processes causing high PPPs concentrations in the drainage network (spills, runoff, drift intercepted by maintenance holes or preferential flow into tile drains).

### 3.4 Acute exposure assessment

Concerning concentrations were measured during large and small events. In the latter, few PPPs exceeded their AQS (i.e., azoxystrobin on June 21^st^) or achieved their maximum concentration and approached their AQS (i.e., napropamide on June 22^nd^). These values could only be quantified because of the continuous high temporal resolution. The 3.5-days time-composite sampling scheme underestimated these maximum values by at least one order of magnitude (Table 3 summarised the PPPs that exceeded their AQS, Figure S15 depicted the variability in underestimation factors for all compounds calculated as the ratio between the maximum concentration of *MS^2^Field* measurements and the time-averaged concentration of NAWA-Trend over the same 3.5-days interval, Figure S16 showed the comparison between time averaged concentrations over 3.5 days calculated from *MS^2^Field* and measured in NAWA-Trend, with corresponding uncertainties in Figure S17). The highest underestimation factors corresponded to PPPs showing high short-term peaks above LOQ values. Depending on the starting time of the composite sampling, the underestimation factors could potentially achieve a value of almost 180 for the 3.5-days scheme (Dax et al., 2020) (Figure S18).

**Table 3.** Measured maximum concentrations during AQS exceedances according to *MS*^*2*^*Field* (Max. conc. *MS*^*2*^*Field*) and measured composite concentrations in the Swiss monitoring program (NAWA-Trend). *: Calculated composite concentration as mean concentration of *MS*^*2*^*Field* measurements over the corresponding 3.5-days interval used in NAWA-Trend for compounds not measured in NAWA Trend in that period (concentration values < LOQ set to LOQ and the neglected missing samples for quality checks purposes amounted to ≈10% within the time intervals). NA stands for PPPs not measured by *MS*^*2*^*Field*. Concentrations at 20 minutes and 3.5 days resolution compared against AQS (exceedances in bold) and RAC (exceedances underlined).

Compound	Time stamp	Max. conc. *MS^2^Field* at 20 min (ng/l)	NAWA-Trend at 3.5 days (ng/l)	Underestimation factor for 3.5-days (-)	AQS (ng/l)	RAC (ng/l)
Azoxystrobin	July 1^st^	**≈6300**	≈490	12.8	550	3300
Diuron	July 7^th^	**≈490**	<LOQ (15)	>32.6	250	1830
Fluopyram	July 1^st^	**≈30,900**	≈2690	11.4	25,100	13,500
Nicosulfuron	July 1^st^	**≈280**	*≈32	*8.8	230	230
Thiacloprid	July 7^th^	**≈2280**	**≈270**	8.3	80	200
Carbendazim	May 2^nd^ - May 6^th^	NA	**≈790**	–	700	–
Chlorpyrifos-methyl	May 9^th^ - May 13^th^	NA	**≈10**	–	7.3	30

*MS^2^Field* revealed a total of 11 exceedances of AQS included in Swiss legislation (WPO, 1998) by 5 PPPs (those in Table 3), while NAWA-Trend captured 3 AQS exceedances by 2 PPPs (azoxystrobin and thiacloprid) during the *MS^2^Field* campaign. The high temporal resolution allowed for the detection of compounds that were missed by NAWA-Trend because of their short peaks, which led to average concentrations below their LOQ (Table 3). Noteworthy were the herbicides mecoprop and diuron, which achieved concentrations as high as ≈610 ng/l and ≈490 ng/l, respectively, the latter exceeding its AQS. Despite urban areas covered less than 1% of the catchment size, we could not rule out the possibility that such peaks resulted from uses in urban areas as herbicides and biocides (Bucheli et al., 1998; Paijens et al., 2020). Short-term peaks occurred during both large and small events. NAWA-Trend measured an exceedance by carbendazim between May 2^nd^ and May 6^th^. Carbendazim was not reapproved as PPP, it was approved as biocide in building materials and it is the major TP of the fungicide thiophanate-methyl approved for orchards among other uses; thiophanate-methyl was applied in the catchment on May 1^st^ and May 2^nd^, supporting the hypothesis that carbendazim resulted from quick transformation of its parent compound. While it was not the goal of this study, we found that a water quality assessment based on chronic exposure only (where 14-days composite samples are compared against chronic quality standards) revealed 2 chronic exceedances during the same 14 days when 5 acute exceedances occurred, thus underestimating the ecotoxicological risk (Table S9). The concentration underestimation factor exceeded 500 for 14-days composite samples (maximum concentration of *MS^2^Field* measurements over the 14-days average concentration of NAWA-Trend in the same interval) (Figure S19). Relevantly for the PPPs regulatory framework, *MS^2^Field* captured 9 exceedances of the regulatory acceptable concentration (RAC) corresponding to 4 PPPs (Table 3), while the standard monitoring recorded 1 RAC exceedance for thiacloprid.

Neither AQS nor RAC exceedances were observed during the dry period (focus of the companion manuscript).

## 4 Discussions

### 4.1 Positive matrix factorisation

We used the PMF to mine our large dataset. Although PMF successfully identified explainable factors, we could not attribute the factors to the contamination sources without uncertainties. In fact, many human-driven decisions influence the processes happening in agricultural catchments including the choice of which, when and where a PPP is applied, which also imply different management of the same land use in the catchment. Regulations also drive what cannot be applied to prevent the spark of resistance to PPPs or to safeguard natural resources. The dynamics in the agricultural landscape, such as crop rotation, imply the use of different PPPs on the same parcel from one year to the other. All these aspects hamper the formation of contamination source areas with unique fingerprints, which would allow for the robust use of PMF to identify them.

### 4.2 Temporal dynamics in a small agricultural stream

*MS^2^Field* enabled to achieve high temporal resolution throughout the entire monitoring period. This allowed for capturing for the first time the concentration patterns of current and legacy PPPs and TPs within and across rain events in a small stream. Our measurements clearly revealed the occurrence of concentration peaks leading or highly lagging water level peaks; more importantly the patterns were inconsistent across events and hardly explainable considering the small size of the catchment. Therefore, there was not a robust proxy for capturing the concentration peaks with proxy-triggered autosamplers. From the coupling of *MS^2^Field* data with the detailed land use map, we could speculate that the further the influential contamination source the longer the arrival of concentration peaks. This expected result was indeed found in another study given the availability of detailed application records relative to the monitored compounds (Lefrancq et al., 2017). The results further demonstrated that compound properties did not have a clear effect on compounds mobilization to the outlet. This was in line with Worrall & Kolpin (2004), who concluded that molecular descriptors found to be significant for aquifer vulnerability to herbicides occurrence were semi-empirical descriptors of molecular topology, such as molecular size, molecular branching and functional group composition rather than typically used properties, such as adsorption and partition coefficients. Nonetheless, our result did not imply that properties were irrelevant to the magnitude of the measured concentrations. Yet, given the high similarities among concentration dynamics of few PPPs, we hypothesized that they originated from the same source and shared the same flow path, as observed in previous controlled studies (Gomides Freitas et al., 2008). Note that, if we measured less targets at a lower frequency, we would not have been able to exploit the observation of practically identical dynamics among compounds to infer on potential contamination sources of several PPPs based on their shared land uses. The more plausible flow paths were through surface runoff over impervious surfaces and drainage network for which it is less likely the physical and chemical properties play a role in the temporal dynamics given the lack of interactions with the soil matrix. While this catchment may sound unique given the dense drainage network, it was estimated that in Switzerland on average 28% of the agricultural area is directly connected to surface water and another 35% is indirectly connected through artificial structures (Schönenberger & Stamm, 2021).

### 4.3 Concentrations in tile drains and runoff

It is generally acknowledged that delays in the order of hours between discharge peaks and concentration peaks can occur, with quick discharge response times being driven by road runoff (Doppler et al., 2012). In our study, we detected several short concentration peaks, with concentrations increasing up to a factor of 10, after small rainfall events (delays up to 8 hours). While we could not find a conclusive explanation for such long delays for this small catchment, the sharp peaks pointed to the regular occurrence of losses from hard surfaces such as roads and farmyards. Hydraulic shortcuts as recently described (Schönenberger & Stamm, 2021) may play an important role in these contexts. Indeed, local observations revealed substantial concentrations in tile drains. Noteworthy was the finding of oxadixyl in tile drain D6 only at concentrations higher than at the catchment outlet by one order of magnitude. This result, in combination with the peaking of oxadixyl in the rising limb of water level (Fig. 4b), indicated that tile drains could deliver pre-event water into surface water. A similar finding was observed and reviewed by Klaus et al. (2013). Regarding this aspect, we became aware of relatively large areas outside from the topographical catchment but connected to the stream through the drainage network. It could be of utmost importance to be aware of the presence of these infrastructures to understand their influence on site-specific target mitigation strategies.

### 4.4 Acute exposure assessment

NAWA-Trend represents an example for national monitoring of PPPs in surface water with continuous relatively high sampling frequency and the inclusiveness of small agricultural streams (<10 km^2^ catchment size). In other European countries grab samples are collected every third week and small streams are excluded. Although, it is the practise to compare the 3.5-days composite samples against the AQS derived from exposure tests lasting between 1 and 4 days, it has been long recognised that active and passive sampling integrating extended periods of time strongly underestimate acute concentration peaks. From an ecotoxicological point of view, this may be critical because short events down to exposure of 30 minutes can be concerning for aquatic health (Ashauer & Brown, 2013). It was also shown with toxicokinetic and toxicodynamic modeling studies that the exposure history (steady or pulsed patterns) affects the survival of sensitive species especially when organisms depurate slowly (Ashauer et al., 2016). At the same time, poorly resolved monitoring may hamper the possibility to causally link the chemical and the biological status of streams. Our dataset provided new quantitative estimates to which degree acute exposure to PPPs may be underestimated by laboratory tests enforced in regulatory studies (exposures typically between 1 and 4 days) (Figure S18). Here, we do not take into account the “cocktail effect” potentially leading to worse detrimental implications when PPPs and TPs with similar mode of actions on non-target organisms are present at the same time also below their AQS.

The raw data generated by HRMS/MS would also allow for suspect screening based on an exact mass and non-target screening using no previous information (Hollender et al., 2017; Krauss et al., 2010). The raw data thus serve as a digital archive, which can be mined to expand risk assessments (Carpenter et al., 2019; Creusot et al., 2020). This is of practical importance because concerns about compounds may rise at later stages, after samples were discarded. For example, we could include key TPs as soon as their corresponding AQS become available to enhance the robustness of risk calculations (Mahler et al., 2021). Despite a broad spectrum of analytes currently implemented in *MS^2^Field*, we could not measure pyrethroids, thus leading to a probable underestimation of the acute risk to non-target organisms including invertebrates (Rosch et al., 2019; Schulz et al., 2021).

*MS^2^Field* made it possible to carry out an accurate but unintended “post-vigilance” monitoring activity of the ensemble of PPPs applied in the catchment. These activities have been urged by several authors to account for landscape-scale factors possibly neglected by the regulatory frameworks and resulting in poor protectiveness of regulatory exposure assessment under real conditions (Knabel et al., 2012; Knauer, 2016; Milner & Boyd, 2017; Schäfer et al., 2019; Vijver et al., 2017). While NAWA-Trend showed that the regulatory framework failed to protect the aquatic environment once for 1 PPP, instead *MS^2^Field* highlighted 9 exceedances of RAC for 4 PPPs. Thanks to continuous progress in analytical chemistry technology we are now able to acquire a comprehensive picture of exposure patterns. Trust and open collaboration among stakeholders are the next steps to effectively reduce the concerning exposure to PPPs in aquatic environments through an in-depth understanding of site-specific predictable criticalities, which indeed are being used to derive risk maps (Koch & Prasuhn, 2021).

### 4.5 Sources identification

#### Availability of public data on agricultural management

Strong collaborative framework for data sharing between farmers and research institutes can accelerate the transition to agriculture sustainability (Della Chiesa et al., 2019). Lacking crucial information such as where, when, how and which compound was applied prevented any attempt to link the high-frequency measurements with contamination sources, the possibility to identify points of risk within parcels (e.g., hydraulic shortcuts) or to exclude possibly not active sources and flowpaths to the stream. Here, vegetables and berry fields are also grown under plastic tunnels (Figure S20), where runoff is prevented and any analysis involving the timing between PPPs application and rainfall to mechanistically understand mobilization to the stream would be wrong. Thus, to fully profit from the cutting-edge analytical workflow, concentration time-series need to be accompanied with spatial and temporal information on relevant field-specific information on agricultural activities. We demonstrated that combining parcel-specific crop information only with general information of possible PPP use based on permitted use from registration could not provide the information for linking observed PPPs peaks in the stream with their sources in the catchment.

#### Target sampling campaigns

With the goal of identifying sources, a spatial sampling campaign over the catchment would be an appropriate approach. Here, we showed that biased proxy-triggered auto samplers could miss leading and lagging concentration peaks. Thus, a continuous monitoring would be needed. Passive samplers may provide the solution for spatially distributed continuous monitoring at a relatively low cost. Yet, while one should consider the research goal when planning for the duration of the sampling, one should also follow good sense in small streams where low water depths may not suffice for a complete immersion of the sampler.

## 5 Conclusion

• The unprecedented continuous high-frequency measurements of 60 catchment-specific compounds (PPPs and TPs) in a small agricultural stream enabled to:
  - Capture a large diversity of unexpected concentration patterns over different events
  - The concentration dynamics were strongly impacted by the application history of the compounds and revealed no obvious relationships with physical-chemical properties;
  - Observe that proxy-triggered auto-sampling can lead to severe biases in measuring concentration peaks as well as gaining a comprehensive picture of exposure patterns in large and small events;
  - Reveal that current monitoring approaches relying on 3.5-days composite samples underestimated the number of exceedances of the legal acute risk and regulatory acceptable concentrations for protecting aquatic organisms during large and small events.
• The positive matrix factorisation analysis discerned (dis)similarities among high-frequency concentration time-series. Uncertainty remained for characterizing influential contamination sources, very likely because it was difficult to extract land use-specific fingerprints considering that the same PPPs can be used on a range of land uses and also because the same land use can be found at different locations of the catchment;
• The high concentrations measured in tile drains and in runoff in the first 20 minutes of water flow pointed to the relevance of these flowpaths in degrading water quality. Yet, high concentrations of a legacy fungicide in one tile drain suggested that this could be the fast flowpath for pre-event water;
• The drainage networks expanded the areas contributing to PPPs losses outside the topographical catchment. Plastic tunnels and hydraulic shortcuts may had influenced the measured time-series by affecting source areas and flowpaths;
• The novel capabilities of *MS^2^Field* highlighted unexpected and overlooked patterns, which could be mechanistically understood only if spatial and temporal information on human interventions are known (PPPs application, irrigation, plastic covers, etc…).

## Data availability

Data presented in this article will be available at https://opendata.eawag.ch/group under the project NAWA-Flowpath.

## Declaration of Competing Interest

The authors declare that they have no known competing financial interests or personal relationships that could have appeared to influence the work reported in this paper.

## Appendix Supplementary materials

Image, application 1

## References

[bib0001] Ashauer R., Albert C., Augustine S., Cedergreen N., Charles S., Ducrot V., Preuss T.G. (2016). Modelling survival: exposure pattern, species sensitivity and uncertainty. Sci. Rep..

[bib0002] Ashauer R., Brown C.D. (2013). Highly time-variable exposure to chemicals–toward an assessment strategy. Integr Environ Assess Manag.

[bib0003] Baldwin A.K., Corsi S.R., Oliver S.K., Lenaker P.L., Nott M.A., Mills M.A., Paatero P. (2020). Primary Sources of Polycyclic Aromatic Hydrocarbons to Streambed Sediment in Great Lakes Tributaries Using Multiple Lines of Evidence. Environ. Toxicol. Chem..

[bib0004] Biggs J., von Fumetti S., Kelly-Quinn M. (2016). The importance of small waterbodies for biodiversity and ecosystem services: implications for policy makers. Hydrobiologia.

[bib0005] Brown S.G., Eberly S., Paatero P., Norris G.A. (2015). Methods for estimating uncertainty in PMF solutions: examples with ambient air and water quality data and guidance on reporting PMF results. Sci. Total Environ..

[bib0006] Bucheli T.D., Müller S.R., Voegelin A., Schwarzenbach R.P. (1998). Bituminous Roof Sealing Membranes as Major Sources of the Herbicide (R,S)-Mecoprop in Roof Runoff Waters: Potential Contamination of Groundwater and Surface Waters. Environ. Sci. Technol..

[bib0007] Carpenter C.M.G., Wong L.Y.J., Johnson C.A., Helbling D.E. (2019). Fall Creek Monitoring Station: Highly Resolved Temporal Sampling to Prioritize the Identification of Nontarget Micropollutants in a Small Stream. Environ. Sci. Technol..

[bib0008] Creusot N., Casado-Martinez C., Chiaia-Hernandez A., Kiefer K., Ferrari B.J.D., Fu Q., Hollender J. (2020). Retrospective screening of high-resolution mass spectrometry archived digital samples can improve environmental risk assessment of emerging contaminants: A case study on antifungal azoles. Environ. Int..

[bib0009] Curchod L., Oltramare C., Junghans M., Stamm C., Dalvie M.A., Roosli M., Fuhrimann S. (2020). Temporal variation of pesticide mixtures in rivers of three agricultural watersheds during a major drought in the Western Cape, South Africa. Water Res X.

[bib0010] Dax A., Stravs M., Stamm C., Ort C., la Cecilia D., Singer H. (2020). MS2Field: Mikroverunreinigungen mobil messen. Zeitlich hochaufgelöste messungen zeigen realistisches ausmass akuter gewässerbelastungen. Aqua & GAS.

[bib0011] Della Chiesa S., la Cecilia D., Genova G., Balotti A., Thalheimer M., Tappeiner U., Niedrist G. (2019). Farmers as data sources: Cooperative framework for mapping soil properties for permanent crops in South Tyrol (Northern Italy). Geoderma.

[bib0012] Doppler T., Camenzuli L., Hirzel G., Krauss M., Lück A., Stamm C. (2012). Spatial variability of herbicide mobilisation and transport at catchment scale: insights from a field experiment. Hydrology and Earth System Sciences.

[bib0013] Doppler T., Dietzel A., Wittmer I., Grelot J., Rinta P., Kunz M. (2020). Mikroverunreinigungen im gewässermonitoring. Ausbau von NAWA TREND und erste resultate 2018. Aqua & GAS.

[bib0014] Gomides Freitas L., Singer H., Muller S., Schwarzenbach R., Stamm C (2008). Source area effects on herbicide losses to surface waters—A case study in the Swiss Plateau. Agriculture, Ecosystems & Environment.

[bib0015] Gustavsson M., Kreuger J., Bundschuh M., Backhaus T. (2017). Pesticide mixtures in the Swedish streams: Environmental risks, contributions of individual compounds and consequences of single-substance oriented risk mitigation. Sci. Total Environ..

[bib0016] Halbach K., Moder M., Schrader S., Liebmann L., Schafer R.B., Schneeweiss A., Reemtsma T. (2021). Small streams-large concentrations? Pesticide monitoring in small agricultural streams in Germany during dry weather and rainfall. Water Res..

[bib0017] Hollender J., Schymanski E.L., Singer H.P., Ferguson P.L. (2017). Nontarget Screening with High Resolution Mass Spectrometry in the Environment: Ready to Go?. Environ. Sci. Technol..

[bib0018] Kiefer K., Du L., Singer H., Hollender J. (2021). Identification of LC-HRMS nontarget signals in groundwater after source related prioritization. Water Res..

[bib0019] Klaus J., Zehe E., Elsner M., Külls C., McDonnell J.J. (2013). Macropore flow of old water revisited: experimental insights from a tile-drained hillslope. Hydrology and Earth System Sciences.

[bib0020] Knabel A., Stehle S., Schafer R.B., Schulz R. (2012). Regulatory FOCUS surface water models fail to predict insecticide concentrations in the field. Environ. Sci. Technol..

[bib0021] Knauer K. (2016). Pesticides in surface waters: a comparison with regulatory acceptable concentrations (RACs) determined in the authorization process and consideration for regulation. Environ Sci Eur.

[bib0022] Koch U., Prasuhn V. (2021). Risikokarten für den Eintrag von Pflanzenschutzmitteln in Oberflächengewässer auf Einzugsgebietsebene. Agroscope Science.

[bib0023] Krauss M., Singer H., Hollender J. (2010). LC-high resolution MS in environmental analysis: from target screening to the identification of unknowns. Anal. Bioanal. Chem..

[bib0024] Lefrancq M., Jadas-Hecart A., La Jeunesse I., Landry D., Payraudeau S. (2017). High frequency monitoring of pesticides in runoff water to improve understanding of their transport and environmental impacts. Sci. Total Environ..

[bib0025] Leu C.S.Heinz, Stamm Christian, Müller Stephan R., Schwarzenbach René P (2004). Variability of Herbicide Losses from Fields to Surface Water within a Small Catchment after a Controlled Herbicide Application. Environ. Sci. Technol..

[bib0026] Lewis K.A., Tzilivakis J., Warner D., Green A. (2016). An international database for pesticide risk assessments and management. Human and Ecological Risk Assessment: An Iqnternational Journal.

[bib0027] Liess M., Liebmann L., Vormeier P., Weisner O., Altenburger R., Borchardt D., Reemtsma T. (2021). Pesticides are the dominant stressors for vulnerable insects in lowland streams. Water Res..

[bib0028] Lorenz S., Rasmussen J.J., Süß A., Kalettka T., Golla B., Horney P., Schäfer R.B. (2016). Specifics and challenges of assessing exposure and effects of pesticides in small water bodies. Hydrobiologia.

[bib0029] Mahler B.J., Nowell L.H., Sandstrom M.W., Bradley P.M., Romanok K.M., Konrad C.P., Van Metre P.C. (2021). Inclusion of Pesticide Transformation Products Is Key to Estimating Pesticide Exposures and Effects in Small U.S. Streams. Environ. Sci. Technol..

[bib0030] Miège C., Mazzella N., Allan I., Dulio V., Smedes F., Tixier C., Vrana B. (2015). Position paper on passive sampling techniques for the monitoring of contaminants in the aquatic environment – Achievements to date and perspectives. Trends in Environmental Analytical Chemistry.

[bib0031] Milner A.M., Boyd I.L. (2017). Toward pesticidovigilance. Can lessons from pharmaceutical monitoring help to improve pesticide regulation?. Science.

[bib0032] Moschet C., Vermeirssen E.L., Singer H., Stamm C., Hollender J. (2015). Evaluation of in-situ calibration of Chemcatcher passive samplers for 322 micropollutants in agricultural and urban affected rivers. Water Res..

[bib0033] Norris G.D.R., Brown S., Bai S (2014). EPA Positive Matrix Factorization (PMF) 5.0 Fundamentals and User Guide.

[bib0034] Paatero P.T., Unto (1994). Positive matrix factorization: a non-negative factor model with optimal utilization of error estimates of data values. Environmetrics.

[bib0035] Paijens C., Bressy A., Frere B., Moilleron R. (2020). Biocide emissions from building materials during wet weather: identification of substances, mechanism of release and transfer to the aquatic environment. Environ. Sci. Pollut. Res. Int..

[bib0036] Polissar A.V., Hopke P.K., Paatero P., Malm W.C., Sisler J.F. (1998). Atmospheric aerosol over Alaska: 2. Elemental composition and sources. Journal of Geophysical Research: Atmospheres.

[bib0037] Popp J., Pető K., Nagy J. (2013). Pesticide productivity and food security. A review. Agronomy for Sustainable Development.

[bib0038] Rosch A., Beck B., Hollender J., Singer H. (2019). Picogram per liter quantification of pyrethroid and organophosphate insecticides in surface waters: a result of large enrichment with liquid-liquid extraction and gas chromatography coupled to mass spectrometry using atmospheric pressure chemical ionization. Anal. Bioanal. Chem..

[bib0039] Schäfer R.B., Liess M., Altenburger R., Filser J., Hollert H., Roß-Nickoll M., Scheringer M. (2019). Future pesticide risk assessment: narrowing the gap between intention and reality. Environmental Sciences Europe.

[bib0040] Schönenberger U., Stamm C. (2021). Hydraulic shortcuts increase the connectivity of arable land areas to surface waters. Hydrology and Earth System Sciences.

[bib0041] Schreiner V.C., Link M., Kunz S., Szocs E., Scharmuller A., Vogler B., Schafer R.B. (2021). Paradise lost? Pesticide pollution in a European region with considerable amount of traditional agriculture. Water Res..

[bib0042] Schulz R., Bub S., Petschick L.L., Stehle S., Wolfram J. (2021). Applied pesticide toxicity shifts toward plants and invertebrates, even in GM crops. Science.

[bib0043] Spycher S., Mangold S., Doppler T., Junghans M., Wittmer I., Stamm C., Singer H. (2018). Pesticide Risks in Small Streams-How to Get as Close as Possible to the Stress Imposed on Aquatic Organisms. Environ. Sci. Technol..

[bib0044] Stehle S., Knabel A., Schulz R. (2013). Probabilistic risk assessment of insecticide concentrations in agricultural surface waters: a critical appraisal. Environ. Monit. Assess..

[bib0045] Stravs M.A., Stamm C., Ort C., Singer H. (2021). Transportable Automated HRMS Platform “MS2field” Enables Insights into Water-Quality Dynamics in Real Time. Environmental Science & Technology Letters.

[bib0046] Strobl R.O., Robillard P.D. (2008). Network design for water quality monitoring of surface freshwaters: a review. J. Environ. Manage..

[bib0047] Ulbrich I.M.C.M.R., Zhang Q., Worsnop D.R., Jimenez J.L (2009). Interpretation of organic components from Positive Matrix Factorization of aerosol mass spectrometric data. Atmos. Chem. Phys..

[bib0048] Vijver M.G., Hunting E.R., Nederstigt T.A., Tamis W.L., van den Brink P.J., van Bodegom P.M. (2017). Postregistration monitoring of pesticides is urgently required to protect ecosystems. Environ. Toxicol. Chem..

[bib0049] Wohl E. (2017). The significance of small streams. Frontiers of Earth Science.

[bib0050] Worrall F., Kolpin D.W. (2004). Aquifer vulnerability to pesticide pollution—combining soil, land-use and aquifer properties with molecular descriptors. Journal of Hydrology.

[bib0051] WPO (1998). 814.201: Waters Protection Ordinance (WPO) of 28 October 1998 (Status as of 1 January 2021), The Swiss Federal Council.

[bib0052] Yang B., Zhou L., Xue N., Li F., Li Y., Vogt R.D., Liu B. (2013). Source apportionment of polycyclic aromatic hydrocarbons in soils of Huanghuai Plain, China: comparison of three receptor models. Sci. Total Environ..

