# Supplementary material for: Continuous high-frequency pesticide monitoring to observe the unexpected and the overlooked
Source: Water Res X. 2021 Nov 7;13:100125. doi: 10.1016/j.wroa.2021.100125 (PMC8593654; doi:10.1016/j.wroa.2021.100125)
Supplement: Supplementary file 1 [file mmc1.docx]

**Supporting information to**

Continuous high-frequency pesticide monitoring to observe the unexpected and the overlooked

D. la Cecilia^1^, A. Dax^1^, H. Ehmann^2^, M. Koster^2^, H. Singer^1^, and C. Stamm^1^

1. Eawag: Swiss Federal Institute of Aquatic Science and Technology, Dübendorf, Switzerland
2. Cantonal Office for the Environment, Thurgau, 8510 Frauenfeld, Switzerland

Corresponding author: Dr. Daniele la Cecilia

Department of Environmental Chemistry

Eawag

8600 Dübendorf, Switzerland

daniele.lacecilia@eawag.ch

**Analytics of MS^2^Field**

Table S1: Analytics by MS^2^Field. Compounds in bold were quantified with an isotope labeled internal standard (ISTD) that had the nearest retention time. The limit of detection was not determined. The workflow of MS^2^Field does not allow for the proper estimation of a measurement error (i.e., measuring multiple times the same sample). Relative recovery not calculated for compounds found below LOQ. Compounds not quantifiable due to poor calibration curves.

| **Name** | **Class** | **In samples** | **LOQ (ng/l)** | **Relative Recovery (%)** | **ISTD** |
| --- | --- | --- | --- | --- | --- |
| **Acetamiprid** | Insecticide | Found | 10 | 48 | Metamitron D5 |
| Atrazine | Herbicide | Found below LOQ | 8 |  | Atrazin D6 |
| **Atrazine-desethyl-desisopropyl** | Metabolite | Found | 15 | 90 | Atrazine-Desethyl 15N3 |
| Azoxystrobin | Fungicide | Found | 5 | 139 | Azoxystrobin D4 |
| **Azoxystrobin free acid** | Metabolite | Found | 20 | 87 | Azoxystrobin D4 |
| Bentazon | Herbicide | Not found | 20 |  | *Bentazon D6* |
| Benzotriazol | Corrosion inhibitor | Found below LOQ | 80 |  | Benzotriazol D4 |
| Boscalid | Fungicide | Not found | 10 |  | *Boscalid D4* |
| **Bupirimate** | Fungicide | Not found | 5 |  | *Metamitron D5* |
| Chloridazon | Herbicide | Found below LOQ | 15 |  | Chloridazon D5 |
| **Chlortoluron** | Herbicide | Not found | 20 |  | Pyrimethanil D5 |
| Clothianidin | Insecticide | Found | 15 | 76 | Clothianidin D3 |
| **Cyproconazol** | Fungicide | Found below LOQ | 35 |  | Epoxiconazol D4 |
| Cyprodinil | Fungicide | Found | 10 | 61 | Cyprodinil D5 |
| Diazinon | Insecticide | Not found | 10 |  | Diazinon D10 |
| **Difenoconazole** | Fungicide | Found | 65 | 91 | Metconazol D6 |
| Dimethenamide | Herbicide | Found | 15 | 67 | Dimethenamid D5 |
| **Dimethoate** | Insecticide | Found | 15 | 70 | Chloridazon D5 |
| Diuron | Herbicide | Found | 6 | 79 | Diuron D6 |
| Epoxiconazole | Fungicide | Found | 15 | 76 | Epoxiconazol D4 |
| **Ethofumesat** | Herbicide | Not found | 40 |  | Isoproturon D6 |
| Fenhexamid | Herbicide | Found | 4 | 74 | Fenhexamid D3 |
| **Fenpyrazimin** | Fungicide | Found | 15 | 66 | Myclobutanil D4 |
| Fluopyram | Herbicide | Found | 4 | 84 | Fluopyram D4 |
| **Flusilazol** | Fungicide | Not found | 40 |  | Epoxiconazol D4 |
| Haloxyfop | Herbicide | Not found | 20 |  | Haloxyfop D4 |
| Imidacloprid | Insecticide, caustic | Not found | 20 |  | Imidacloprid D4 |
| **Iprodione** | Fungicide | Not quantifiable | 6 |  | Boscalid D4 |
| **Iprovalicarb** | Fungicide | Not found | 10 |  | Epoxiconazol D4 |
| Isoproturon | Herbicide | Not found | 10 |  | Isoproturon D6 |
| **Kresoxim-methyl** | Fungicide | Found below LOQ | 35 |  | Epoxiconazole D4 |
| **Lenacil** | Herbicide | Not found | 20 |  | Diuron D6 |
| Mecoprop | Herbicide | Found | 4 | 78 | Mecoprop D6 |
| Metalaxyl | Fungicide | Not quantifiable | 50 |  | Metalaxyl D6 |
| Metamitron | Herbicide | Found | 5 | 85 | Metamitron D5 |
| **Methoxyfenozide** | Insecticide | Found | 15 | 68 | Dimethenamid D5 |
| Metolachlor | Herbicide | Found | 4 | 73 | Metolachlor D6 |
| Metribuzin | Herbicide | Found | 70 | 77 | Metribuzin D3 |
| **Monuron** | Herbicide | Not found | 10 |  | Thiacloprid D4 |
| Myclobutanil | Fungicide | Found | 3 | 81 | Myclobutanil D4 |
| **Napropamide** | Herbicide | Found | 7 | 77 | Fluopyram D4 |
| **Neotam** | Sweetener | Not found | 15 |  | Metalaxyl D6 |
| **Nicosulfuron** | Herbicide | Found | 30 | 112 | Simazin D5 |
| DEET | Insect repellent | Found | 8 | 77 | N-N-diethyl-3-methylbenzamid (DEET) D10 |
| **Oxadixyl** | Fungicide | Found | 6 | 107 | Thiacloprid D4 |
| **Penconazole** | Fungicide | Found | 15 | 80 | Diazinon D10 |
| **Pirimicarb** | Insecticide | Not quantifiable | 2 |  | Thiacloprid D4 |
| Propamocarb | Fungicide | Not found | 20 |  | Propamocarb free base D6 |
| **Propiconazole** | Fungicide | Found | 6 | 74 | Diazinon D10 |
| Propyzamide | Herbicide | Not found | 2 |  | Propyzamide D3 |
| Pyrimethanil | Fungicide | Found | 4 | 74 | Pyrimethanil D5 |
| Simazine | Herbicide | Found | 2 | 104 | Simazin D5 |
| **Simeton** | Herbicide | Not quantifiable | 4 |  | Imidacloprid D4 |
| Tebuconazole | Fungicide | Found | 6 | 75 | Tebuconazole D9 |
| **Tebufenozide** | Insecticide | Found below LOQ | 30 |  | Tebuconazole D9 |
| Terbutylazine | Herbicide | Found | 2 | 76 | Terbutylazine D5 |
| **Terbutylazin-desethyl** | Herbicide | Found | 3 | 69 | Atrazin D5 |
| Thiacloprid | Insecticide | Found | 8 | 68 | Thiacloprid D4 |
| **Thiamethoxam** | Insecticide | Found below LOQ | 100 |  | Clothianidin D3 |
| **Trifloxystrobin** | Fungicide | Not quantifiable | 100 |  | Metconazol D6 |

**Analytics by Interkantonales Labor, Schaffhausen**

Samples were analyzed using direct injection liquid chromatography coupled to mass spectrometry (*Table S2*). The target list included 93 compounds and metabolites (Table S3). Samples were centrifuged and a solution containing isotope-labeled internal standards (ISTD) was spiked. Randomly selected samples were spiked with a standard solution in order to assess relative recovery of the compounds. An injection volume of 120 µL was used and chromatographic separation was performed (Agilent Zorbax Eclipse XDB C18, 2.1 x 100 mm, 1.8 µm), applying a water-methanol gradient (both containing 0.1% formic acid/formate buffer). The measurements were performed on a triple quadrupole mass spectrometer (Agilent TripleQuad 6490A) after electrospray ionization acquiring at least to MRM transitions (quantifier and qualifier) per compound. Quantification of the target compounds was performed using the Masshunter Software (Agilent). LOQ values were in the range of 7 – 40 ng/l with an uncertainty ranging between 10% – 35% (Table S3).

*Table S2: Details of the method used by Interkantonales Labor, Schaffhausen.*

| **Internal Method Number** | **M-0473** | **M-0462** | **M-0488** |
| --- | --- | --- | --- |
| Internal Method  Version | 1 | 3 | 1 |
| Sample preparation | centrifuge | centrifuge | centrifuge |
| Sample enrichment | no | no | no |
| Injection volume/ µl | 120 | 120 | 120 |
| LC System | Agilent Infinity 1290 | Agilent Infinity 1290 | Agilent Infinity 1290 |
| MS System | Agilent TripleQuad 6490A | Agilent TripleQuad 6490A | Agilent TripleQuad 6490A |
| Quantification Software | Agilent Masshunter | Agilent Masshunter | Agilent Masshunter |
| LC column | Agilent Zorbax Eclipse XDB C18, 2.1 x 100 mm, 1.8µm | Agilent Zorbax SB-Aq, 2.1 x 100 mm, 1.8 µm | Waters Acquity UPLC BEH C18, 130 Å, 2.1 × 75 mm, 1.7 μm |
| Eluent A | water + 0.1% formic acid /formate buffer | water + 0.5% formic acid | water + 0.1% formic acid /formate buffer |
| Eluent B | methanol + 0.1% formic acid /formate buffer | methanol + 0.5% formic acid | methanol + 0.1% formic acid /formate buffer |
| Isotop-labeled internal standard used | yes | yes | yes |
| Independent reference material used | yes | yes | yes |
| Relative recovery checked | yes | yes | yes |

Table S3: Analytics by Interkantonales Labor, Schaffhausen. We are not for which compound the corresponding isotope labeled standards was used for quantification.

| **Internal Method Number** | Parameter | Limit of  quantification (µg/l) | Uncertainty (%) |
| --- | --- | --- | --- |
| M-0473 | 1H-Benzotriazol | 0.04 | 10% |
|  | 4-/5-Methylbenzotriazol | 0.015 | 10% |
|  | Atenolol | 0.012 | 10% |
|  | Atrazin | 0.035 | 15% |
|  | Azithromycin | 0.034 | 25% |
|  | Azoxystrobin | 0.025 | 15% |
|  | Bezafibrat | 0.016 | 25% |
|  | Boscalid | 0.033 | 20% |
|  | Carbamazepin | 0.033 | 10% |
|  | Carbendazim | 0.017 | 10% |
|  | Chloridazon | 0.007 | 10% |
|  | Chloridazon-desphenyl | 0.015 | 10% |
|  | Chlorpyrifos | 0.027 | 15% |
|  | Chlortoluron | 0.011 | 10% |
|  | Clarithromycin | 0.03 | 20% |
|  | Cyproconazol | 0.018 | 15% |
|  | Cyprodinil | 0.026 | 25% |
|  | DEET (Diethyltoluamid) | 0.016 | 30% |
|  | Diazinon | 0.013 | 10% |
|  | Diclofenac | 0.02 | 15% |
|  | Dimethoat | 0.012 | 10% |
|  | Diuron | 0.015 | 15% |
|  | Epoxiconazol | 0.016 | 15% |
|  | Erythromycin | 0.02 | 30% |
|  | Erythromycin-H2O | 0.015 | 25% |
|  | Ethofumesat | 0.033 | 20% |
|  | Imidacloprid | 0.014 | 10% |
|  | Iprovalicarb | 0.024 | 15% |
|  | Isoproturon | 0.025 | 15% |
|  | Linuron | 0.007 | 10% |
|  | Mefenaminsäure | 0.015 | 10% |
|  | Metalaxyl | 0.015 | 15% |
|  | Metamitron | 0.019 | 10% |
|  | Metazachlor | 0.009 | 15% |
|  | Metformin | 0.034 | 20% |
|  | Methoxyfenozid | 0.011 | 15% |
|  | Metolachlor | 0.017 | 15% |
|  | Metoprolol | 0.009 | 10% |
|  | Metribuzin | 0.015 | 10% |
|  | Napropamid | 0.016 | 15% |
|  | Naproxen | 0.029 | 25% |
|  | Nicosulfuron | 0.011 | 35% |
|  | Pirimicarb | 0.014 | 10% |
|  | Propamocarb | 0.022 | 10% |
|  | Pyrimethanil | 0.02 | 20% |
|  | Sotalol | 0.017 | 10% |
|  | Sulfamethazin | 0.013 | 10% |
|  | Sulfamethoxazol | 0.01 | 15% |
|  | Tebuconazol | 0.014 | 10% |
|  | Terbuthylazin | 0.014 | 20% |
|  | Terbutryn | 0.009 | 10% |
|  | Thiacloprid | 0.009 | 10% |
|  | Thiamethoxam | 0.008 | 10% |
|  | Trimethoprim | 0.028 | 15% |
| M-0462 | 2,4-D | 0.02 | 30% |
|  | Acesulfam-K | 0.04 | 30% |
|  | Azoxystrobinsäure | 0.02 | 30% |
|  | Bentazon | 0.02 | 30% |
|  | Chlorothalonil Metabolit R417888 | 0.02 | 30% |
|  | Dichlorprop | 0.04 | 30% |
|  | Dimethenamid-ESA | 0.02 | 30% |
|  | Dinoseb | 0.02 | 30% |
|  | MCPA | 0.02 | 30% |
|  | Mecoprop | 0.02 | 30% |
|  | Mesotrion | 0.02 | 30% |
|  | Metazachlor ESA | 0.04 | 30% |
|  | Metolachlor-ESA | 0.04 | 20% |
|  | Metolachlor-OXA | 0.04 | 20% |
|  | Propachlor-ESA | 0.02 | 20% |
|  | Propachlor-OXA | 0.04 | 20% |
|  | Sulcotrion | 0.04 | 30% |
| M-0488 | Acetamiprid | 0.022 | 25% |
|  | Bupirimat | 0.025 | 25% |
|  | Chlorantraniliprol | 0.022 | 20% |
|  | Clothianidin | 0.029 | 25% |
|  | Diflufenican | 0.016 | 20% |
|  | Dimethachlor | 0.017 | 20% |
|  | Dimethenamid | 0.017 | 25% |
|  | Fenhexamid | 0.02 | 20% |
|  | Fenoxycarb | 0.026 | 25% |
|  | Fenpyrazamin | 0.021 | 30% |
|  | Fludioxonil | 0.04 | 30% |
|  | Flufenacet | 0.019 | 25% |
|  | Fluopyram | 0.014 | 25% |
|  | Foramsulfuron | 0.019 | 35% |
|  | Haloxyfop | 0.024 | 25% |
|  | Mesosulfuron-methyl | 0.03 | 25% |
|  | Methiocarb | 0.031 | 30% |
|  | Methomyl | 0.027 | 25% |
|  | Propiconazol | 0.017 | 20% |
|  | Propyzamid | 0.009 | 20% |
|  | Spiroxamin | 0.018 | 30% |
|  | Tebufenozid | 0.02 | 25% |

**Analytics by InterLabor, Belp**

The water sample is filtered through a paper filter and the pH adjusted to 7. With each series, a blank sample (tap water) and a recovery sample (spiked tap water at 0.1 μg/l) is prepared and analysed. An aliquot of 250 ml of each sample is used for further sample preparation. A solution of several isotope-labeled internal standards (ISTD) is added to each sample.

- Sample preparation for determination of pesticides (higher polarity / higher masses) by LC-MS/MS

For solid phase extraction (SPE) (Oasis HLB 3 CC, 60 mg), the cartridges are conditioned with 3 ml of methanol and 3 ml of pure water prior to slowly pass the 250 ml aliquote of each sample. The cartridges are brought to dryness with suction and subsequently eluated with 3 ml of methanol. The collected eluate in a PE-tube is evaporated to dryness (TurboVap, 45 °C). The residues are brought back into solution with 500 μl of acetonitrile. Extract is transferred to a vial for HPLC injection. Standard solutions of 200 ng/ml and 20 ng/ml in acetonitrile are prepared for external calibration of the system.

- Sample preparation for determination of pesticides (lower polarity / lower masses) by GC-MS/MS

The 250 mL aliquote is extracted with a first quantity of 8 ml dichloromethane (DCM) in a separating funnel. The DCM is collected and the extraction is repeated with a second quantity of 8 ml of DCM. The DCM of both extraction steps are combined and dried with little sodium sulfate. In a PE-tube, the DCM is evaporated to dryness (TurboVap, 45 °C). The residues are brought back into solution with 5 ml of 0.05 % (v/v) formic acid in acetonitrile. 100 μl are transferred to a vial for GC analysis (higher concentrated analytes). The remaining solution again evaporated to dryness (TurboVap, 45 °C). The residues are brought back into solution with 500 μl of 0.05 % (v/v) formic acid in acetonitrile and transferred to second vial for GC analysis (lower concentrated analytes). Standard solutions of 200 ng/ml and 20 ng/ml in acetonitrile are prepared for external calibration of the system.

- Analysis of water samples

The prepared solutions are analysed by liquid chromatography with tandem mass spectrometry (LC-ESI-MS/MS) and gas chromatography with tandem mass spectrometry (GC-EI-MS/MS). Each positive signal is verified by the signal ratio of two or more mass fragments. This allows obtaining results with a high specificity. In addition, the preparation and analysis of a spiked water sample and the usage of internal standards are helpful to control the accuracy of the results. In combination of both techniques some 500 compounds are covered.

- System and components

HPLC: Agilent Technologies, HPLC 1290 Infinity

MS/MS: Sciex, API 6500+

ESI source: Sciex, Ion Drive Turbo V Ion Source with TurboIonSpray

HPLC column: Phenomenex Aqua, 75 x 2.0 mm, 3 μm, 125 Å (00C-4311-B0)

GC-MS/MS: Agilent Technologies, GC-MS Triple Quad 7000C

GC column: Zebron ZB-Multiresidue-1, 30 m x 0.25 mm, 0.25 μm (7HG-G016-11)

- Limit of quantification

The analytical method is capable of quantifying most of the analytes at a lower limit of 0.01 μg/l (related to the water sample) . Significant detected signals with concentrations below 0.01 μg/l will be reported as “trace”. Some analytes may have a higher limit of quantification due to sensitivity, interferences or partial loss during sample preparation.

In this study, we were not provided with a list of compounds with corresponding LOQ values. Still, we are interested in compounds that were measured at high concentrations.


**Estimation of the measurement error for *MS^2^Field***

The estimatation of the *MS^2^Field* measurement errors per compound exploits the high sampling frequency in dry periods. In fact, we expect that concentration levels may change slowly due to the lack of meteorological drivers, and therefore, differences in concentrations measured in samples collected 20 minutes apart should reflect the measurement error. For this analysis, we select six successive data points collected in each evening of June 28^th^, June 29^th^ and 30^th^ (evening was the period with lower fluctuations of the concentrations as shown in detail in the companion manuscript revealing the presence of diel fluctuations).

As shown in the table below, we calculate one mean concentration using C1, C3 and C5 and another mean concentration using C2, C4 and C6 (three replicas averaging possible trends in concentrations during the two hours. Then, we calculate the absolute median relative % difference. The calculation is repeated using the concentrations measured on June 29^th^ and June 30^th^. Finally, we calculate a mean absolute median relative % difference using the three values for the three days and we keep the maximum value as the final measurement error. This approach is used for compounds with concentrations above the LOQ at all selected times.

| Date and time | Concentration | Mean concentration | Absolute median relative % difference |
| --- | --- | --- | --- |
| 6.28.2019 19:07:53 | C1 | M1=(C1+C3+C5)/3 | \|M2-M1\|/((M2+M1)/2) * 100 |
| 6.28.2019 19:28:00 | C2 |  |  |
| 6.28.2019 19:48:08 | C3 |  |  |
| 6.28.2019 20:08:15 | C4 | M2=(C2+C4+C6)/3 |  |
| 6.28.2019 20:28:23 | C5 |  |  |
| 6.28.2019 20:48:31 | C6 |  |  |

For compounds with concentrations below the corresponding LOQ in at least one selected time, we calculate a measurement error using samples that were spiked with known concentrations of the target compounds during the quality check routine of MS2Field, as shown in the table below. On June 28^th^, 29^th^ and 30^th^ there were three spikes, one per day. To the spiked concentrations, we substracted the concentration measured at the previous time step (because the concentrations are below the LOQ, we substracted a concentration equal to half the LOQ). Then, we calculate an absolute median relative % difference using each combination without repetition of the calculated differences between two days (i.e., 28^th^-29^th^, 28^th^-30^th^, 29^th^-30^th^). Finally, we keep the maximum absolute median relative % difference among the three values for the three combinations.

| Date and time | Concentration before spike | Concentration after spike | Difference | median relative % difference |
| --- | --- | --- | --- | --- |
| 2.28.2019 | C0_A | C1_A | C_A=C1_A-C0_A | \|C_B-C_A\|/((C_B+C_A)/2) * 100 |
| 2.29.2019 | C0_B | C1_B | C_B=C1_B-C0_B |  |

Table S4: Estimated measurement error for MS^2^Field

| Name | Abbreviation | Error |
| --- | --- | --- |
| Acetamiprid | Acetamiprid | 8 |
| Azoxystrobin | Azoxystrobin | 7 |
| Azoxystrobin_free_acid | Azoxystrobin-TP | 4 |
| Clothianidin | Clothianidin | 4 |
| Cyprodinil | Cyprodinil | 11 |
| DEET | DEET | 6 |
| Dimethenamide | Dimethenamide | 3 |
| Diuron | Diuron | 7 |
| Epoxiconazole | Epoxiconazole | 5 |
| Fenhexamid | Fenhexamid | 7 |
| Fenpyrazamin | Fenpyrazamin | 5 |
| Fluopyram | Fluopyram | 3 |
| Mecoprop | Mecoprop | 3 |
| Metamitron | Metamitron | 10 |
| Metolachlor | Metolachlor | 1 |
| Myclobutanil | Myclobutanil | 7 |
| Nicosulfuron | Nicosulfuron | 5 |
| Napropamide | Napropamide | 5 |
| Oxadixyl | Oxadixyl | 4 |
| Pyrimethanil | Pyrimethanil | 3 |
| Simazin | Simazin | 5 |
| Tebuconazole | Tebuconazole | 6 |
| Terbutylazine | Terbutylazine | 9 |
| Terbutylazin_desethyl | Terbutylazin-TP | 6 |
| Thiacloprid | Thiacloprid | 9 |

**Hydrological classification**

Table S5: Summary of the large rain events.

| Date start | Date end | Sum rain over event (mm) | Maximum intensity in the event (mm/10 min) | Average intensity in the event (mm/10 min) | Duration of the event (hours) | Minimum water level in the event (m) | Maximum water level in the event (m) | Standard deviation of the water level in the event (m) |
| --- | --- | --- | --- | --- | --- | --- | --- | --- |
| 28.05.2019 02:20 | 28.05.2019 06:30 | 3.2 | 1.2 | 0.1 | 4.2 | 0.158 | 0.264 | 0.015 |
| 10.06.2019 06:00 | 10.06.2019 07:30 | 1.9 | 1.1 | 0.2 | 1.5 | 0.121 | 0.247 | 0.015 |
| 10.06.2019 10:10 | 10.06.2019 13:30 | 18.2 | 10.4 | 0.9 | 3.3 | 0.121 | 0.315 | 0.022 |
| 10.06.2019 20:00 | 11.06.2019 03:10 | 16.4 | 1.7 | 0.4 | 7.2 | 0.121 | 0.315 | 0.022 |
| 12.06.2019 01:00 | 12.06.2019 07:20 | 22.1 | 1.4 | 0.6 | 6.3 | 0.121 | 0.449 | 0.042 |
| 15.06.2019 19:00 | 16.06.2019 01:00 | 8.3 | 3.8 | 0.2 | 6.0 | 0.149 | 0.449 | 0.052 |
| 01.07.2019 22:20 | 02.07.2019 00:50 | 27.2 | 14.2 | 1.7 | 2.5 | 0.121 | 0.463 | 0.023 |
| 07.07.2019 03:00 | 07.07.2019 05:50 | 6.1 | 1.2 | 0.3 | 2.8 | 0.121 | 0.463 | 0.022 |

Table S6. Summary of the small rain events.

| Date start | Date end | Sum rain over event (mm) | Maximum intensity in the event (mm/10 min) | Average intensity in the event (mm/10 min) | Duration of the event (hours) | Minimum water level in the event (m) | Maximum water level in the event (m) | Standard deviation of the water level in the event (m) |
| --- | --- | --- | --- | --- | --- | --- | --- | --- |
| 27.05.2019 21:30 | 27.05.2019 21:30 | 0.2 | 0.2 | 0.2 | 0.0 | 0.173 | 0.176 | 0.001 |
| 28.05.2019 09:40 | 28.05.2019 10:30 | 0.3 | 0.1 | 0.1 | 0.8 | 0.170 | 0.178 | 0.002 |
| 28.05.2019 21:00 | 28.05.2019 22:40 | 2.1 | 0.5 | 0.2 | 1.7 | 0.163 | 0.212 | 0.017 |
| 29.05.2019 12:30 | 29.05.2019 13:10 | 0.9 | 0.4 | 0.2 | 0.7 | 0.155 | 0.205 | 0.015 |
| 29.05.2019 15:20 | 29.05.2019 15:20 | 0.2 | 0.2 | 0.2 | 0.0 | 0.159 | 0.177 | 0.004 |
| 10.06.2019 17:40 | 10.06.2019 17:40 | 0.1 | 0.1 | 0.1 | 0.0 | 0.149 | 0.168 | 0.005 |
| 15.06.2019 00:00 | 15.06.2019 00:20 | 0.3 | 0.2 | 0.1 | 0.3 | 0.165 | 0.170 | 0.001 |
| 16.06.2019 07:40 | 16.06.2019 07:50 | 0.2 | 0.1 | 0.1 | 0.2 | 0.153 | 0.157 | 0.001 |
| 18.06.2019 18:40 | 18.06.2019 18:40 | 0.2 | 0.2 | 0.2 | 0.0 | 0.147 | 0.150 | 0.001 |
| 19.06.2019 05:00 | 19.06.2019 05:00 | 0.1 | 0.1 | 0.1 | 0.0 | 0.143 | 0.147 | 0.001 |
| 19.06.2019 20:40 | 19.06.2019 20:50 | 0.7 | 0.5 | 0.4 | 0.2 | 0.145 | 0.157 | 0.004 |
| 20.06.2019 04:30 | 20.06.2019 04:30 | 0.1 | 0.1 | 0.1 | 0.0 | 0.142 | 0.164 | 0.006 |
| 20.06.2019 10:50 | 20.06.2019 12:40 | 1.2 | 0.2 | 0.1 | 1.8 | 0.140 | 0.155 | 0.004 |
| 20.06.2019 17:20 | 20.06.2019 17:40 | 1 | 0.8 | 0.3 | 0.3 | 0.146 | 0.169 | 0.006 |
| 21.06.2019 23:00 | 22.06.2019 01:10 | 1.6 | 0.3 | 0.1 | 2.2 | 0.142 | 0.173 | 0.008 |

**Concentrations measured with *MS^2^Field and comparison with NAWA-Trend***


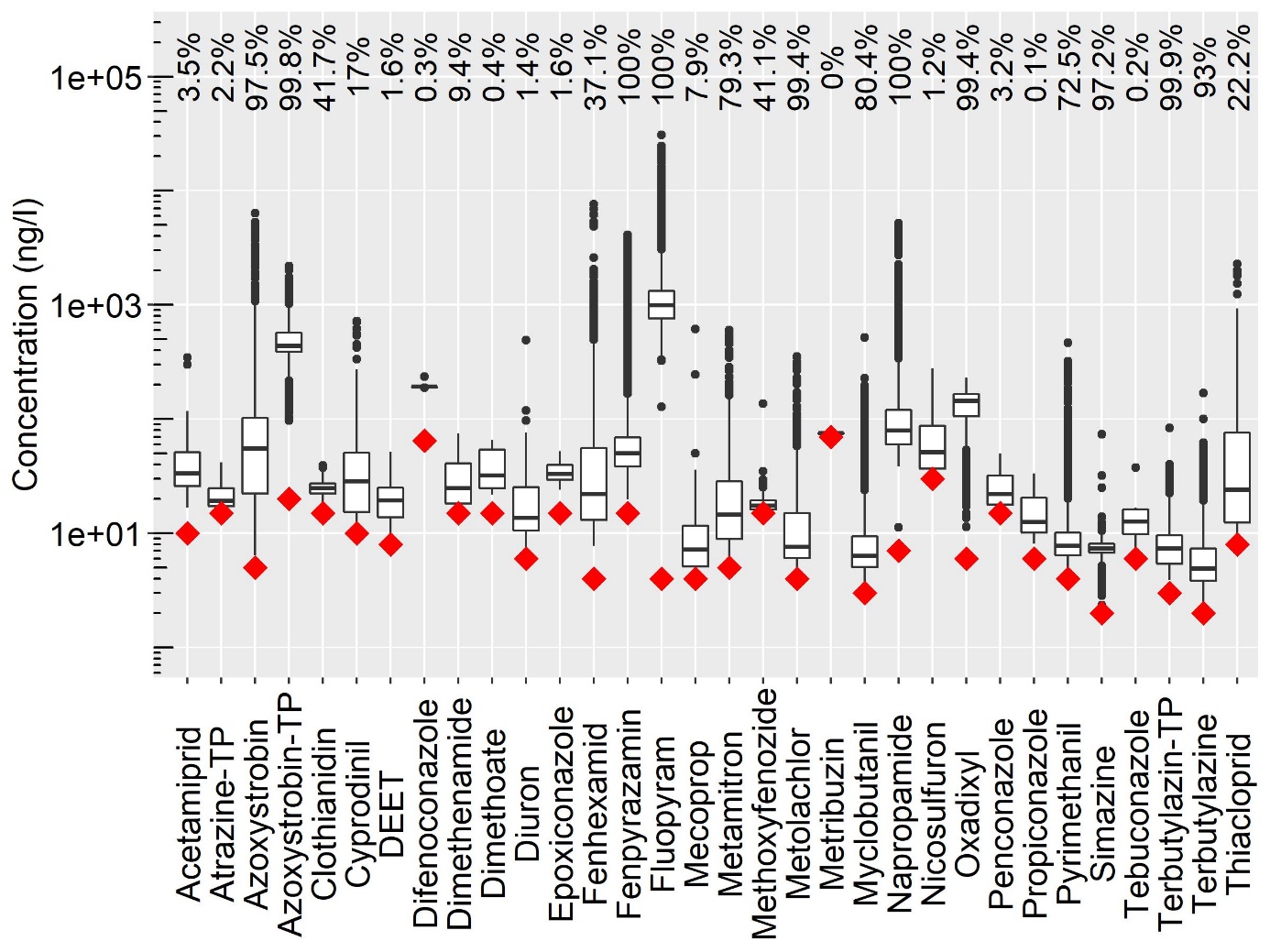


*Figure S1: Concentrations measured with MS^2^Field represented in log10 scale, with values smaller than the corresponding LOQ not shown. LOQ values shown as red diamonds. The percentage of concentration values above LOQ, excluding data gaps, per compound over the monitoring is reported above the boxplots.* *Azoxystrobin-TP corresponds to azoxystrobin free acid and Terbutylazin-TP corrsponds to terbutylazin desethyl.*

Figure S2: Concentration time series of compounds measured by MS^2^Field with concentrations above LOQ (green line and bullets). In black, the time series of the compounds that were also measured by NAWA-Trend. MS^2^Field was used from May 27^th^ to July 7^th^ (light grey background) and NAWA-Trend collected 3.5 days composite samples from April to July. In red, the acute quality standard (AQS). Vertical bars in gold show periods with data gaps due to maintenance of MS^2^Field. Atrazine-TP corresponds to atrazine desethyl desisopropyl and Azoxystrobin-TP corresponds to azoxystrobin free acid.

Figure S3: Concentration time series of compounds measured by MS^2^Field with concentrations above LOQ (green line and bullets). In black, the time series of the compounds that were also measured by NAWA-Trend. MS^2^Field was used from May 27^th^ to July 7^th^ (light grey background) and NAWA-Trend collected 3.5 days composite samples from April to July. In red, the acute quality standard (AQS). Vertical bars in gold show periods with data gaps due to maintenance of MS^2^Field.

Figure S4: Concentration time series of compounds measured by MS^2^Field with concentrations above LOQ (green line and bullets). In black, the time series of the compounds that were also measured by NAWA-Trend. MS^2^Field was used from May 27^th^ to July 7^th^ (light grey background) and NAWA-Trend collected 3.5 days composite samples from April to July. In red, the acute quality standard (AQS). Vertical bars in gold show periods with data gaps due to maintenance of MS^2^Field.

Figure S5: Concentration time series of compounds measured by MS^2^Field with concentrations above LOQ (green line and bullets). In black, the time series of the compounds that were also measured by NAWA-Trend. MS^2^Field was used from May 27^th^ to July 7^th^ (light grey background) and NAWA-Trend collected 3.5 days composite samples from April to July. In red, the acute quality standard (AQS). Vertical bars in gold show periods with data gaps due to maintenance of MS^2^Field. Terbutylazin-TP corresponds to terbutylazin desethyl.

Table S7: List of the maximum concentrations reached by the 43 compounds in common between MS^2^Field and NAWA-TREND. 10 compounds peaked during the MS^2^Field campaign; 13 compounds peaked before the MS^2^Field campaign, 7 peaked afterwards and 13 compounds were below LOQ (not shown).

| *Name* | *Concentration* | *Date_st* | *Date_en* |
| --- | --- | --- | --- |
| *Metalaxyl* | *10* | *22.04.2019 07:00* | *25.04.2019 19:00* |
| *Acetamiprid* | *10* | *25.04.2019 19:00* | *29.04.2019 07:00* |
| *Cyprodinil* | *110* | *02.05.2019 19:00* | *06.05.2019 07:00* |
| *Fenhexamid* | *500* | *02.05.2019 19:00* | *06.05.2019 07:00* |
| *Chloridazon* | *10* | *06.05.2019 07:00* | *09.05.2019 19:00* |
| *Epoxiconazole* | *10* | *09.05.2019 19:00* | *13.05.2019 07:00* |
| *Tebufenozid* | *60* | *09.05.2019 19:00* | *13.05.2019 07:00* |
| *Napropamide* | *1440* | *16.05.2019 19:00* | *20.05.2019 07:00* |
| *Imidacloprid* | *29* | *20.05.2019 07:00* | *23.05.2019 19:00* |
| *Metribuzin* | *31* | *20.05.2019 07:00* | *23.05.2019 19:00* |
| *Ethofumesat* | *110* | *20.05.2019 07:00* | *23.05.2019 19:00* |
| *Metamitron* | *175* | *20.05.2019 07:00* | *23.05.2019 19:00* |
| *Pyrimethanil* | *202* | *20.05.2019 07:00* | *23.05.2019 19:00* |
| *Nicosulfuron* | *19* | *06.06.2019 15:00* | *10.06.2019 03:00* |
| *Pirimicarb* | *19* | *06.06.2019 15:00* | *10.06.2019 03:00* |
| *Fenpyrazamin* | *650* | *06.06.2019 15:00* | *10.06.2019 03:00* |
| *Clothianidin* | *20* | *11.06.2019 07:00* | *14.06.2019 19:00* |
| *Dimethenamide* | *31* | *11.06.2019 07:00* | *14.06.2019 19:00* |
| *Terbutylazine* | *35* | *11.06.2019 07:00* | *14.06.2019 19:00* |
| *Metolachlor* | *183* | *11.06.2019 07:00* | *14.06.2019 19:00* |
| *Thiamethoxam* | *28* | *24.06.2019 06:30* | *27.06.2019 18:30* |
| *Methoxyfenozide* | *12* | *01.07.2019 06:00* | *04.07.2019 18:00* |
| *Thiacloprid* | *274* | *04.07.2019 18:00* | *08.07.2019 06:00* |
| *Diazinon* | *16* | *After July 8^th^* | *After July 8^th^* |
| *Diuron* | *21* | *After July 8^th^* | *After July 8^th^* |
| *DEET* | *82* | *After July 8^th^* | *After July 8^th^* |
| *Mecoprop* | *845* | *After July 8^th^* | *After July 8^th^* |
| *Azoxystrobin* | *1380* | *After July 8^th^* | *After July 8^th^* |
| *Azoxystrobin_free_acid* | *2440* | *After July 8^th^* | *After July 8^th^* |
| *Fluopyram* | *5220* | *After July 8^th^* | *After July 8^th^* |

**Positive Matrix Factorization**

We used a range of factors from 2 to 13 (i.e. the number of good compounds minus 1). The model was able to accurately model most of the compounds using six to nine factors (Figure S6). The evaluation of the ratio between Qrobust (*Q_robust_*) and Qexpected (*Q_exp_*) suggests that increasing the number of factors above six does not lead to a strong decrease in the ratio and above eleven the ratio becomes smaller than 1 indicating overfitting (Figure S7). The quality of the solution with a number of factors higher than 6 is improving possibly simply thank to the higher degrees of freedom introduced by the increasing number of factors accounted for. Finally, we notice that the factor profiles of the solutions with a number of factors above six begin to mimic the time series of the target compounds, while do not predict a superposition of the effects as prescribed by the mathematical framework (Figure S8). Given all the above considerations, we decided to retain the solution with six factors (Table S5).

With the selected number of factors, we performed a bootstrap analysis to analyse the quality of the solution. We used the default parameters provided by the software (i.e., R^2^ = 0.6, block size = 818 data points and number of bootstraps = 100). The mapping between factors and boot factors was generally acceptable because above 70%. The boot factor 2 was matched with factor 1 in less than 10 runs and with factor 5 in less than 20 runs. The non-match could be due to the creation of a block size that does not contain the data points peculiar of the factor. In fact, factors 2 consist of: (1) an early negligible baseline similar to factor 5, (2) a peculiar response to the large events between June 10^th^ and June 12^th^, (3) a noisy baseline similar to factor 1 up to June 23^rd^ and (4) a negligible baseline at the end.


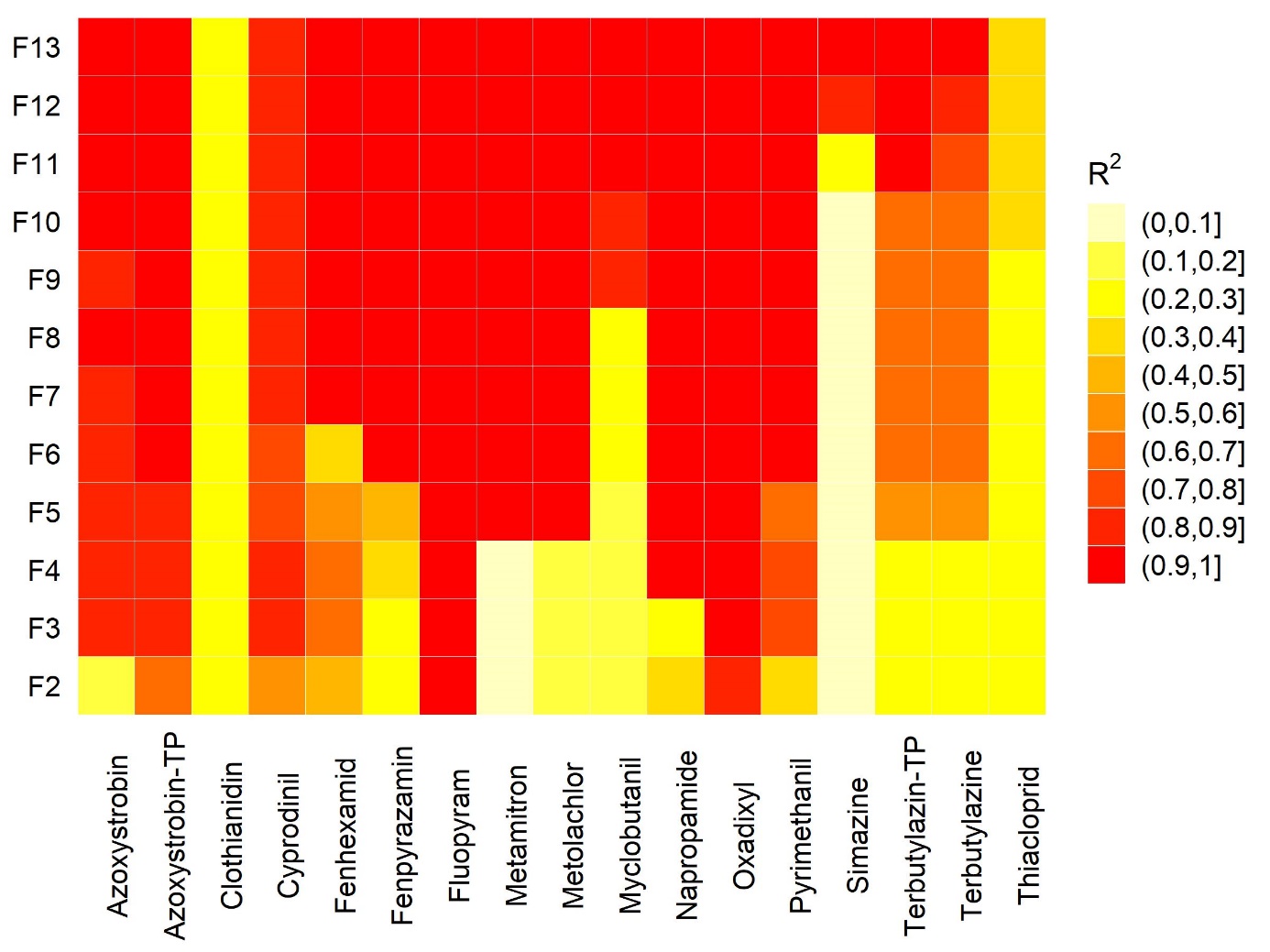


Figure S6: R^2^ between measured concentrations and predicted concentrations by the PMF for the different number of factors accounted for. Azoxystrobin-TP corresponds to azoxystrobin free acid and Terbutylazin-TP corresponds to terbutylazin-desethyl.


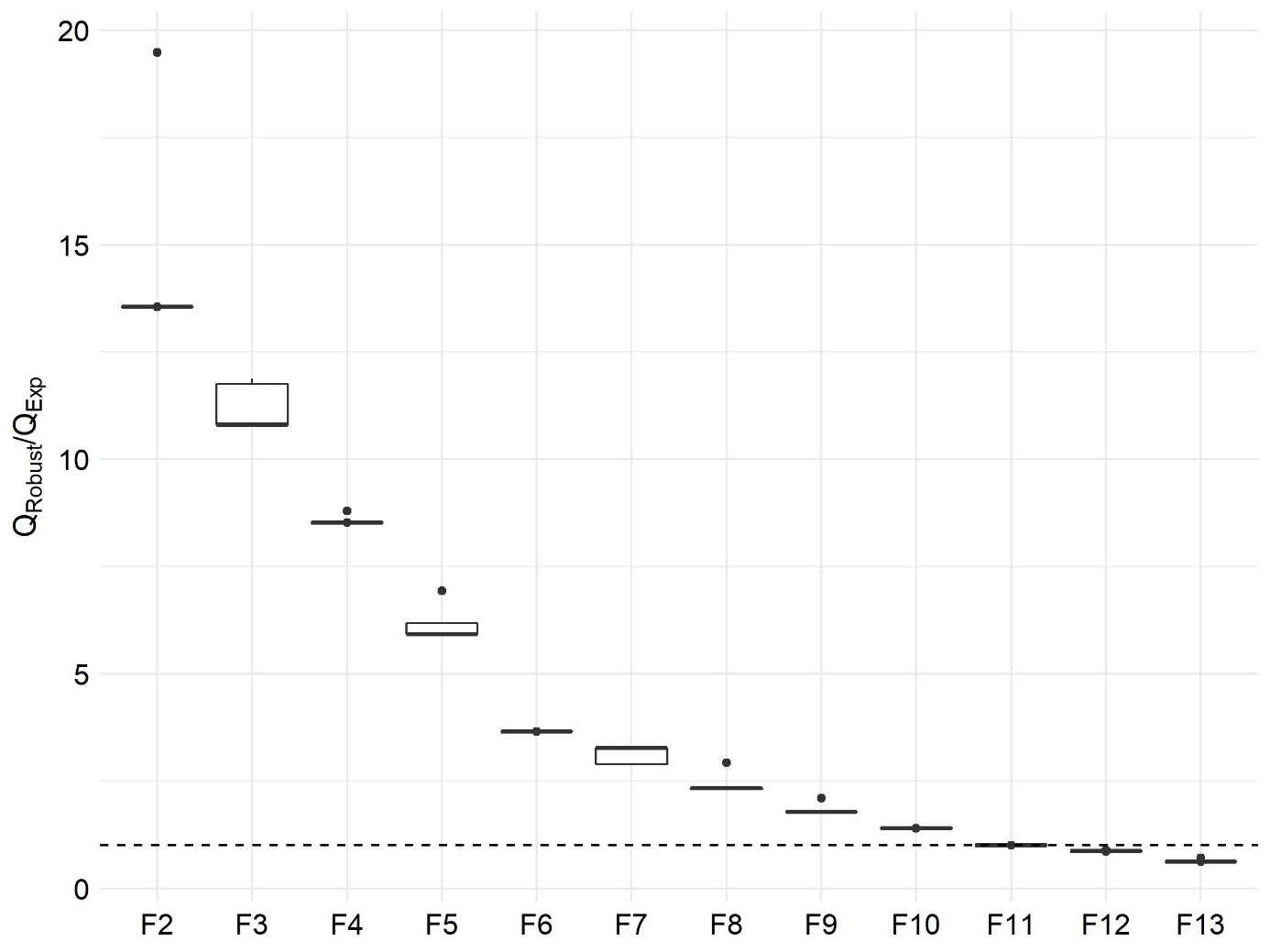


Figure S7: Ratios between Q_robust_ and Q_exp_ for the 20 model runs of each different number of factors accounted for. The dashed line indicates a ratio equal to 1.

Figure S8: Profiles of the factors for each PMF solution with the range of factors accounted for. x-axis is the date in the format “month-day”.

Table S8: Summary of the selected PMF scenario with six factors.

| Name | Class | % above LOQ | R^2^ |
| --- | --- | --- | --- |
| \| Azoxystrobin \| \| --- \| \| Azoxystrobin_free_acid \| \| Clothianidin \| \| Cyprodinil \| \| Fenhexamid \| \| Fenpyrazamin \| \| Fluopyram \| \| Metamitron \| \| Metolachlor \| \| Myclobutanil \| \| Napropamide \| \| Oxadixyl \| \| Pyrimethanil \| \| Simazin \| \| Terbutylazine \| \| Terbutylazin_desethyl \| \| Thiacloprid \| | \| Good \| \| --- \| \| Good \| \| Weak \| \| Weak \| \| Good \| \| Good \| \| Good \| \| Good \| \| Good \| \| Good \| \| Good \| \| Good \| \| Good \| \| Good \| \| Good \| \| Good \| \| Weak \| | \| 97.5 \| \| --- \| \| 99.8 \| \| 41.7 \| \| 17 \| \| 37.1 \| \| 100 \| \| 100 \| \| 79.3 \| \| 99.4 \| \| 80.4 \| \| 100 \| \| 99.4 \| \| 72.5 \| \| 97.2 \| \| 93 \| \| 99.9 \| \| 22.2 \| | \| \| 0.84 \| \| --- \| \| 0.91 \| \| 0.23 \| \| 0.8 \| \| 0.36 \| \| 0.95 \| \| 0.94 \| \| 0.98 \| \| 0.92 \| \| 0.23 \| \| 1 \| \| 0.92 \| \| 0.93 \| \| 0.04 \| \| 0.63 \| \| 0.62 \| \| 0.28 \| \| \| --- \| --- \| --- \| --- \| --- \| --- \| --- \| --- \| --- \| --- \| --- \| --- \| --- \| --- \| --- \| --- \| --- \| --- \| |


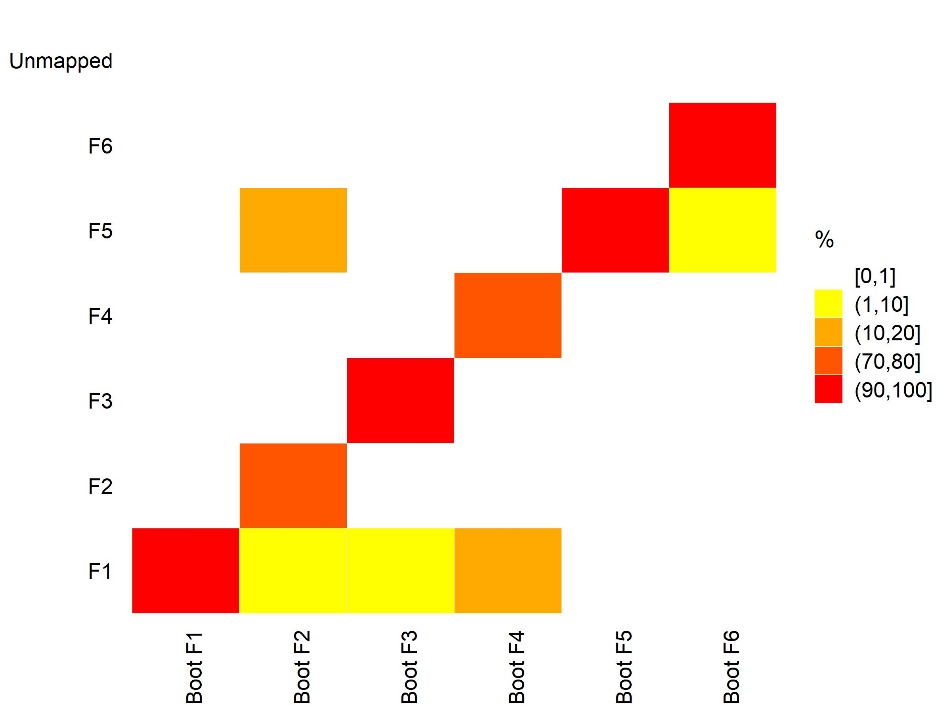


Figure S9: Summary of the bootstrap analysis using the suggested block size of 818 data points and the coefficient of determination R of 0.6. On the x-axis the factors of the bootstrap analysis and on the y-axis the factors of the reference model results.

Concentration patterns of different compounds with different properties in different rain events.

**Large event 1: May 28^th^ 2019, 02:20.**

Figure S10: The legend reports the maximum concentrations of each PPPs achieved during the event; normalised concentration values are represented on the y-axis. Water level as thick black dash line in (a) and (b) and thin line for readability from (c) to (f). Rainfall as black line on the reversed y-axis. Vertical bars in gold show periods with data gaps due to maintenance of MS^2^Field.

**Large events 2-3-4: June 10^th^ 2019, 06:00 - June 10^th^ 2019, 10:10 - June 10^th^ 2019, 20:00 (presented in the main manuscript in Figure 3)**

**Large event 5: June 12^th^ 2019, 01:00 (not captured due to maintenance to *MS^2^Field)***

**Large event 6: June 15^th^ 2019, 19:00**

Figure S11: The legend reports the maximum concentrations of each PPPs achieved during the event; normalised concentration values are represented on the y-axis. Water level as thick black dash line in (a) and (b) and thin line for readability from (c) to (f). Rainfall as black line on the reversed y-axis. Vertical bars in gold show periods with data gaps due to maintenance of MS^2^Field.

**Large event 7: July 1^st^ 2019, 22:20**

Figure S12: The legend reports the maximum concentrations of each PPPs achieved during the event; normalised concentration values are represented on the y-axis. Water level as thick black dash line in (a) and (b) and thin line for readability from (c) to (f). Rainfall as black line on the reversed y-axis. Vertical bars in gold show periods with data gaps due to maintenance of MS^2^Field.

**Large event 6: July 7^th^ 2019, 03:00 (not entirely captured because the monitoring was ended)**

Figure S13: The legend reports the maximum concentrations of each PPPs achieved during the event; normalised concentration values are represented on the y-axis. Water level as thick black dash line in (a) and (b) and thin line for readability from (c) to (f). Rainfall as black line on the reversed y-axis. Vertical bars in gold show periods with data gaps due to maintenance of MS^2^Field.

**Concentrations in tile drains and runoff over 2019**


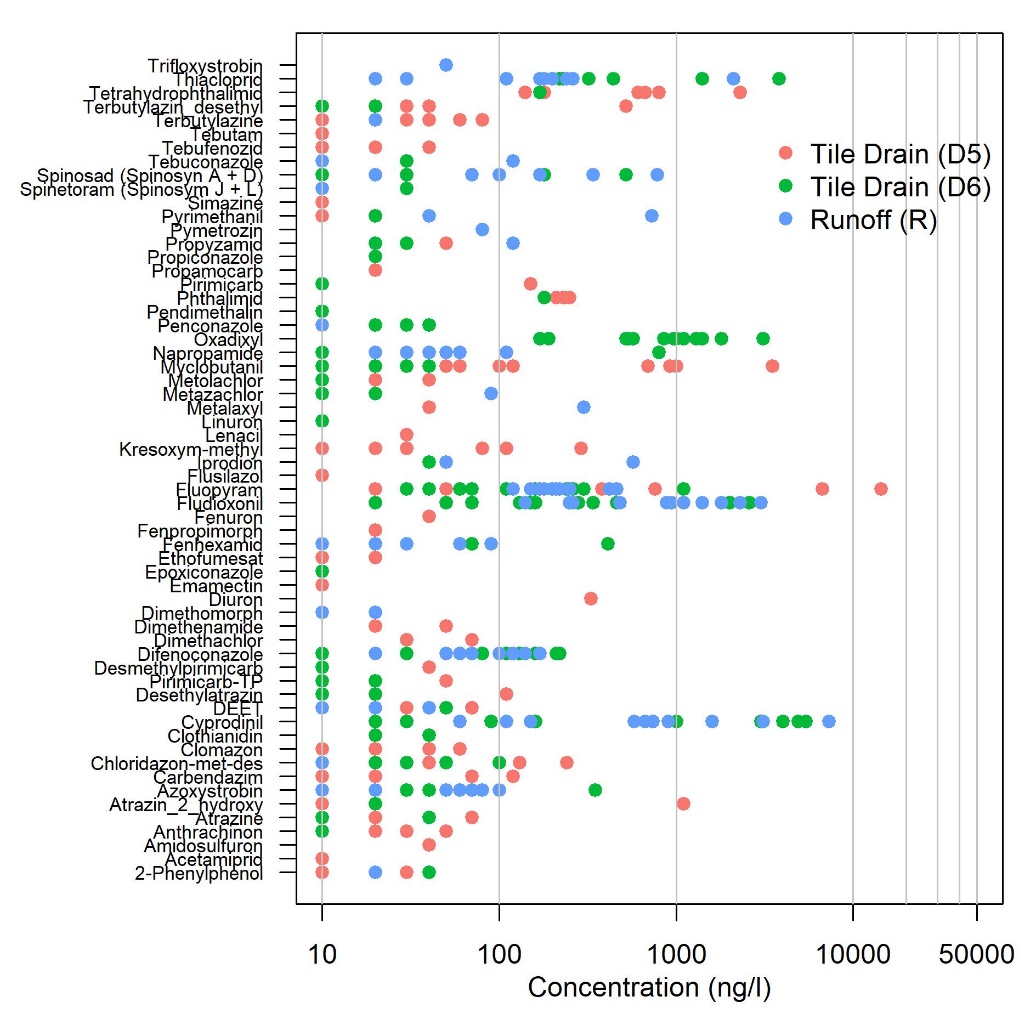


*Figure S14: Concentrations in tile drains and runoff over 2019 (also outside the time window covered with MS^2^Field).*


**Underestimation factor**


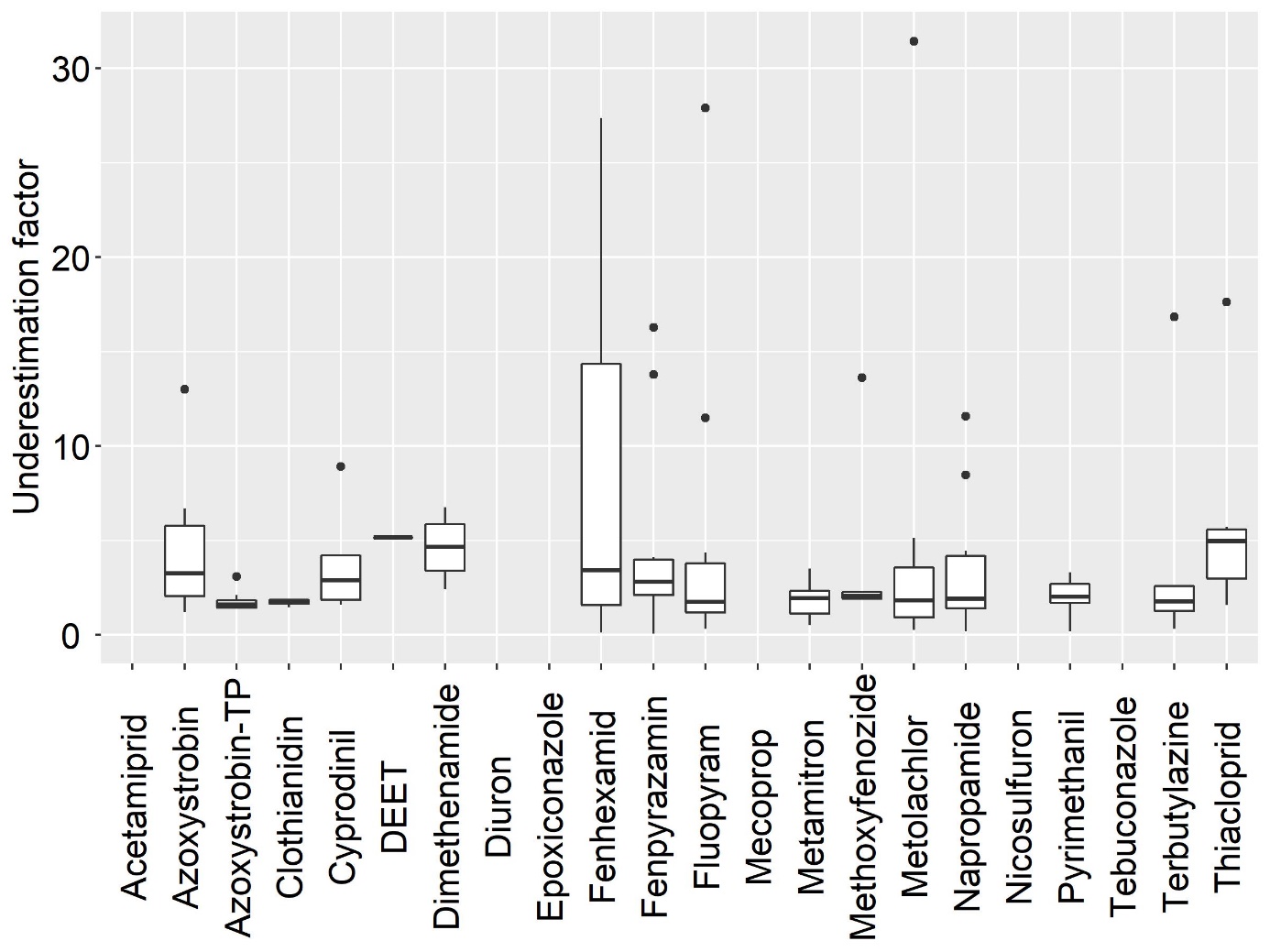
*Figure S15: Underestimation factor calculated as the ratio between the maximum concentration measured by MS^2^Field and the mean concentration measured by NAWA-Trend in the corresponding 3.5-day composite sample, for all the composite samples collected during the MS^2^Field campaign. To calculate MS^2^Field mean concentrations, values below LOQ were set to LOQ and data gaps were disregarded. Azoxystrobin-TP corresponds to azoxystrobin free acid. Compounds with no underestimation factor calculated due to observation of episodic peaks resulting in below LOQ concentratons with extended time composite samples.*

Though, different laboratory procedures may yield differences in measured concentrations. Thus, the underestimation factor calculated in Figure S15 may be biased by the different analytical methods of *MS^2^Field* and the laboratory in Schaffhausen. To highlight potential differences in the measurements, we calculated mean concentrations using the *MS^2^Field* data relative to the 3.5 days corresponding to the time-composite samples of NAWA-Trend and we compared this calculated values with the concentrations measured by NAWA-Trend (Figure S16).


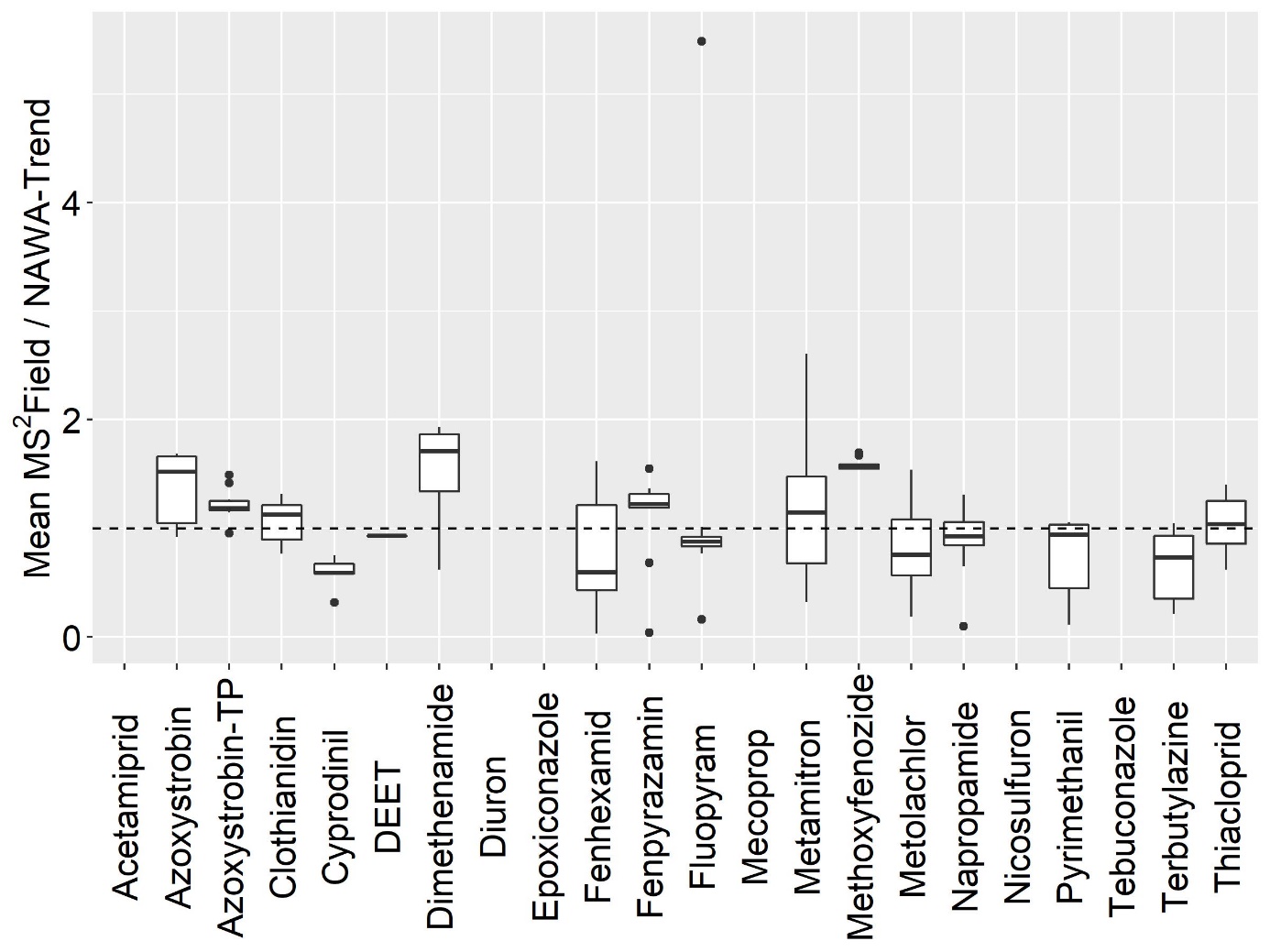


Figure S16: Ratio between the mean concentration calculated using the MS^2^Field measurements and the mean concentration measured by NAWA-Trend in the same 3.5-day composite sample, for all the composite sampled collected during the MS^2^Field campaign, grouped by compound. To calculate MS^2^Field mean concentrations, values below LOQ were set to LOQ and data gaps were disregarded. The dashed line indicates a ratio equal to 1. Azoxystrobin-TP corresponds to azoxystrobin free acid.

As expected, often there were small deviations from the ratio of 1 and in few instances, the deviation was substantial. Thus, we inspected the ratio over time for each compound together with an error propagation analysis. To this aim, we assigned the measurement error quantified by the laboratory in Schaffhausen for each compound to all concentration data points for the corresponding compound measured in the context of NAWA-Trend. The error for each compound measured by *MS^2^Field* was calculated as in Section S4.

When calculating the mean over 3.5 days using *MS^2^Field*, we are summing the measurements and dividing by the number of data points. Being A_i_ the measurement at time t_i_ for a given compound up to *n* with n being the number of times, the sum X=$\sum_{n} A_{n}$ will have an error U_X = $nU\_A$. The division Y= X/n, with Y being the mean over a time window, will have an error U_Y = U_X/n.

Now, Z is the measured concentration in the NAWA-Trend program, with a measured error U_Z.

Finally, the error of the ratio R=Y/Z will have an error U_R = R*(U_Y*Y + U_Z*Z).


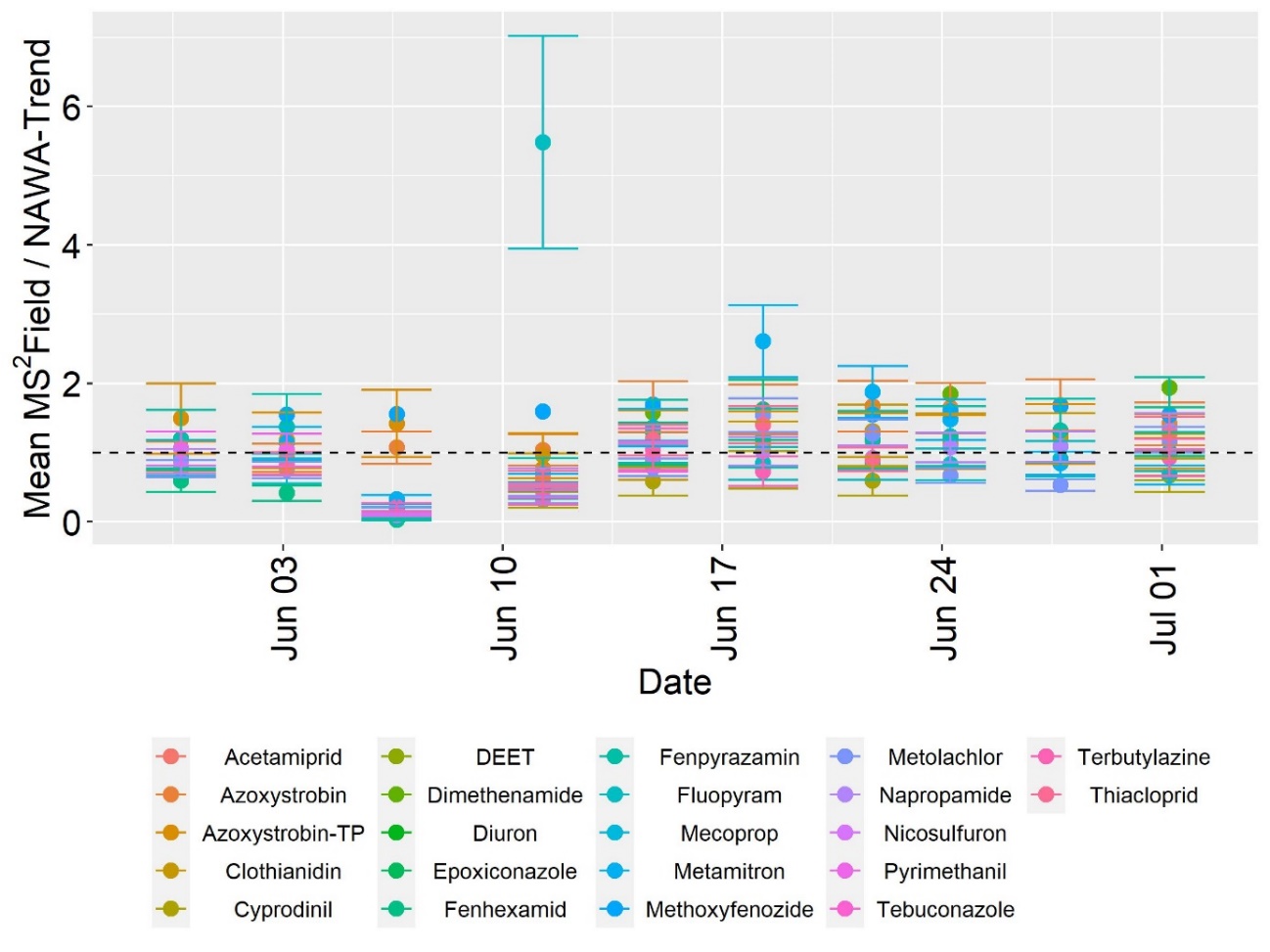


Figure S17: Error propagation analysis of the ratio between the mean concentration calculated using the MS^2^Field measurements and the mean concentration measured by NAWA-Trend in the same 3.5-day composite sample, for all the composite sampled collected during the MS^2^Field campaign, grouped by time. To calculate MS^2^Field mean concentrations, values below LOQ were set to LOQ and data gaps were disregarded. The dashed line indicates a ratio equal to 1. Azoxystrobin-TP corresponds to azoxystrobin free acid.

The error in the measurements can explain the small deviations from a value of the ratio close to 1. Azoxystrobin was systematically overestimated, while cyprodinil was underestimated. This may be due to the corresponding recoveries, of 140% for azoxystrobin and of 60% for cyprodinil. But few substantial deviations remained. This is because in the interval between June 6 and June 10, the NAWA-Trend measurement were largely higher than the calculated mean concentrations of *MS^2^Field*. While relative to June 6, the reason is not known, in June 10, *MS^2^Field* had an issue and did not collect water samples for several hours during a large rain event, which is expected to drive high concentration peaks for several compounds (e.g., fluopyram). The NAWA-Trend measurements indicate that we very likely missed a concerning pollution event.

Acknowledging the limitations highlighted in Section S9, we calculated 3.5-days mean concentrations using the *MS^2^Field* measurements, starting from the beginning of the *MS^2^Field* monitoring campaign. This allowed for:

(i) considering measurements in the period between June 10^th^ at 07:00 and June 11^th^ at 19:00 when NAWA-Trend was not operational;

(ii) Running a sensitivity analysis where we shifted the starting point of the resampling by 20 minutes until a complete shift of 3.5 days was reached to avoid repetitions (note that in this way we discarded concentration data up to 3.5 days after the beginning of *MS^2^Field* monitoring, which however was not a concerning period in terms of concentration peaks).


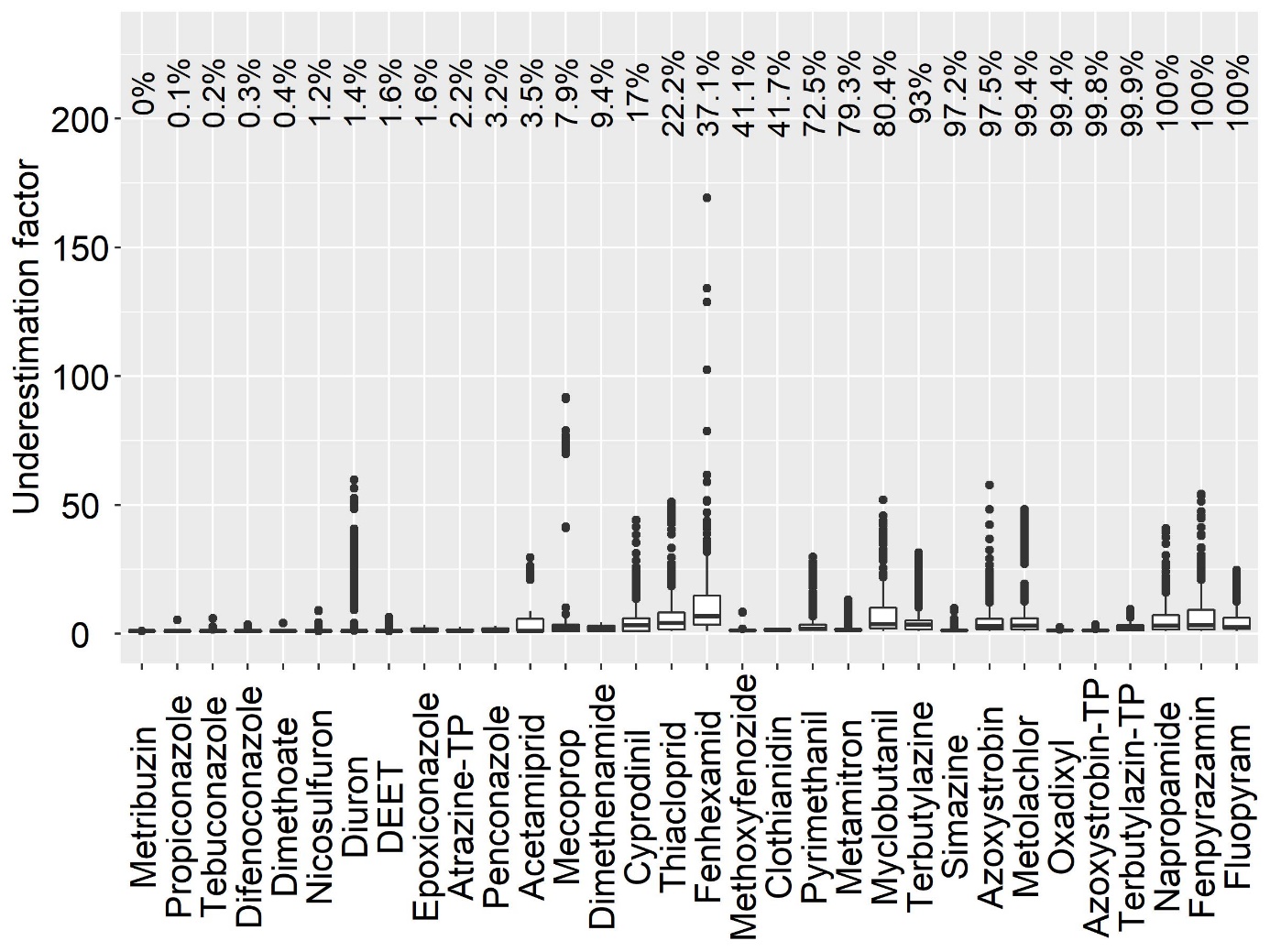


Figure S18: Numerical underestimation factor calculated considering time composite samples of 3.5 days. Compounds sorted by the percentage of detections during the MS^2^Field monitoring (percentages, excluding data gaps, reported above the boxplots). Atrazine-TP corresponds to atrazine desethyl desisopropyl, Azoxystrobin-TP corresponds to azoxystrobin free acid and Terbutylazin-TP corresponds to terbutylazin desethyl.

We repeated the analysis by calculating mean concentrations over 14 days as this is the standard in other stations part of the Swiss national monitoring program.


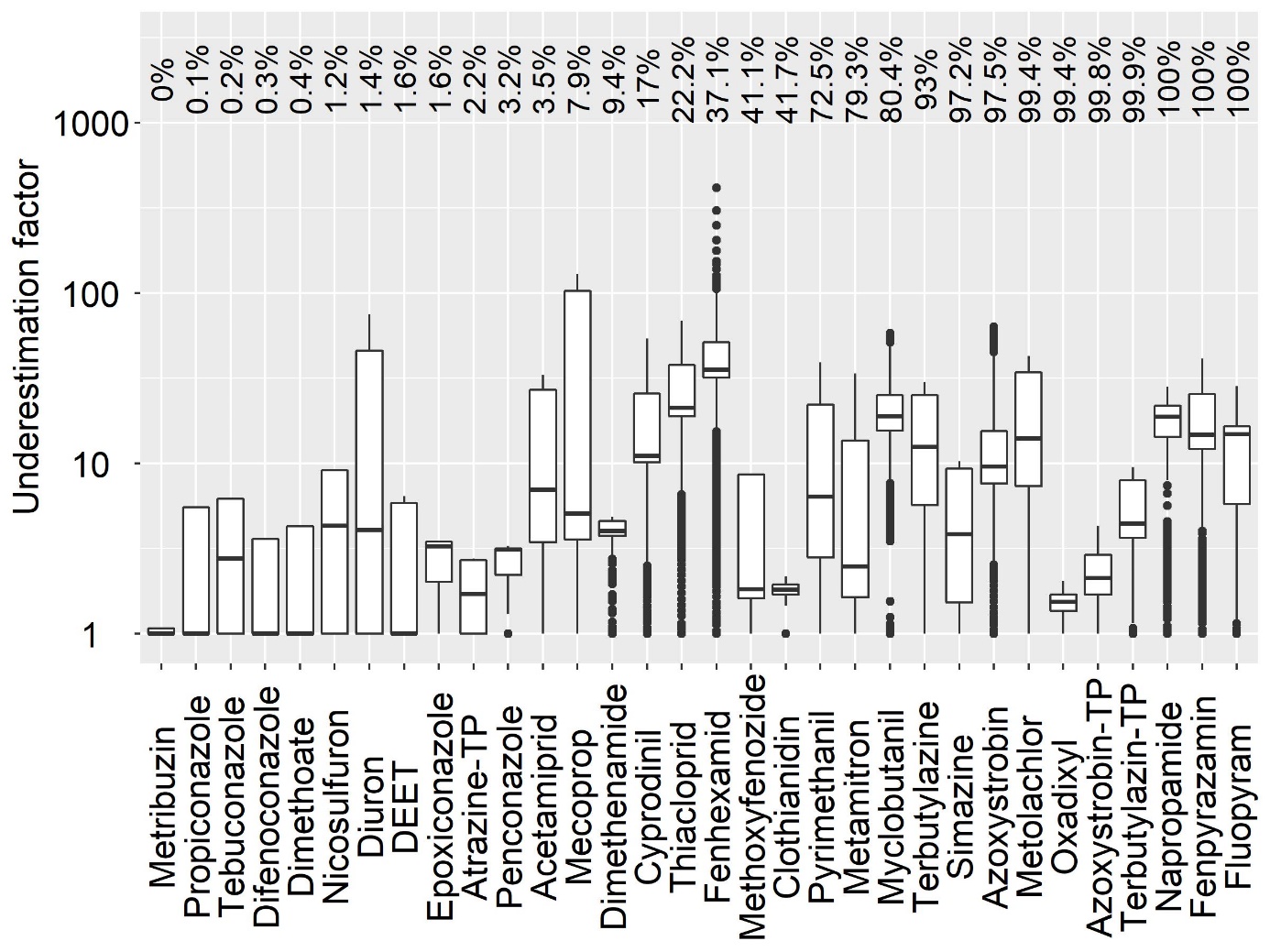


Figure S19: Numerical underestimation factor calculated considering time composite samples of 14 days. Compounds sorted by the percentage of detections during the MS^2^Field monitoring (percentages, excluding data gaps, reported above the boxplots). Atrazine-TP corresponds to atrazine desethyl desisopropyl, Azoxystrobin-TP corresponds to azoxystrobin free acid and Terbutylazin-TP corresponds to terbutylazin desethyl.

**Comprehensive acute and chronic exposure assessment.** We performed the water quality assessment against acute quality standards (AQS) and chronic quality standards (CQS) currently reported in the Swiss legislation. An assessment based on chronic exposure only would had underestimated the risk given that during the 14 days when we registered with *MS^2^Field* the 5 AQS exceedances reported in Table S6, only 2 CQS exceedances occurred. In particular, azoxystrobin exceeded its CQS but not its AQS, while thiacloprid exceeded both standards. We further noted that the chronic exposure to nicolfuron could not be quantified given the high LOQ of both methods (i.e., LOQ by *MS^2^Field* is 30 ng/l and LOQ by NAWA-Trend is 11 ng/l, respectively reported in Table S1 and *Table S2*).

Table S9: Measured maximum concentrations during AQS exceedances according to MS^2^Field (MS^2^Field), measured time composite concentrations of 3.5 days in the Swiss monitoring program (NAWA-Trend 3.5 days) and calculated time composite samples of 14 days using the NAWA-Trend data (NAWA-Trend 14 days). Calculated composite concentration as mean concentration of MS^2^Field measurements over the corresponding 3.5-days interval (indicated with *) and 14-days interval (indicated with **) used in NAWA-Trend for compounds not measured in NAWA Trend in that period (concentration values < LOQ set to LOQ and the neglected missing samples for quality checks purposes amounted to ≈10% within the time intervals). NA stands for PPPs not measured by MS^2^Field. Concentrations at 20 minutes and 3.5 days resolution compared against AQS (pale yellow background), while concentrations at 14 days resolution compared against CQS (light blue background). Quality standards exceedances in bold. ***: LOQ>CQS, therefore the risk assessment was not reliable.

| Compound | Time stamp | *MS^2^Field* (ng/l) | NAWA-Trend 3.5 days  (ng/l) | NAWA-Trend 14-days (ng/l) | AQS  (ng/l) | CQS  (ng/l) | Underestimation factor for 3.5-days  (-) | Underestimation factor for 14-days  (-) |
| --- | --- | --- | --- | --- | --- | --- | --- | --- |
| Azoxystrobin | July 1^st^ | **≈6300** | ≈490 | ≈**360** | 550 | 200 | 12.8 | 17.5 |
| Diuron | July 7^th^ | **≈490** | <LOQ (15) | <LOQ (15) | 250 | 70 | >32.6 | >32.6 |
| Fluopyram | July 1^st^ | **≈30900** | ≈2690 | ≈1860 | 25100 | 13500 | 11.4 | 16.6 |
| Nicosulfuron | July 1^st^ | **≈280** | *≈32 | **≈30 | 230 | 8.7*** | *8.8 | **9.2 |
| Thiacloprid | July 7^th^ | **≈2280** | **≈270** | ≈**78** | 80 | 10 | 8.3 | 28.9 |
| Carbendazim | May 2^nd^ - May 6^th^ | NA | **≈790** | ≈250 | 700 | 440 | - | - |
| Chlorpyrifos-methyl | May 9^th^ - May 13^th^ | NA | **≈10** | ≈**2.5** | 7.3 | 1 | - | - |


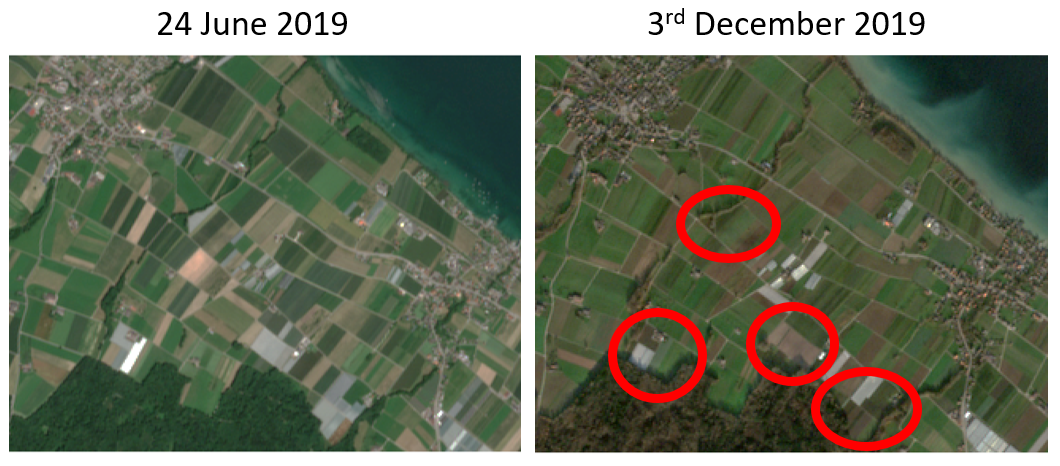


*Figure S20: Images from the satellite Sentinel-2 show the potential for identifying spatial-temporal dynamics of plastic tunnels.*
